# Circulating Tumor DNA in Genitourinary Cancers: Detection, Prognostics, and Therapeutic Implications

**DOI:** 10.3390/cancers16122280

**Published:** 2024-06-20

**Authors:** Margo B. Gerke, Caroline S. Jansen, Mehmet A. Bilen

**Affiliations:** 1 Emory University School of Medicine, Atlanta, GA 30322, USA; mgerke@emory.edu (M.B.G.); carey.jansen@emory.edu (C.S.J.); 2 Winship Cancer Institute of Emory University, Atlanta, GA 30322, USA; 3 Department of Hematology and Medical Oncology, Emory University School of Medicine, Atlanta, GA 30322, USA

**Keywords:** circulating tumor DNA, prostate cancer, bladder cancer, renal cell carcinoma, liquid biopsy, genitourinary cancers, tumor biomarkers

## Abstract

**Simple Summary:**

Circulating tumor DNA (ctDNA) is a non-invasive method of identifying and monitoring genitourinary cancers, including prostate, bladder, and renal cell carcinoma, via blood or urine samples. CtDNA introduces a potential method for cancer screening. If detected, ctDNA may reveal genetic alterations that have prognostic value. As treatment options for genitourinary cancers progress towards accounting for tumor genetic profiles, ctDNA may predict which patients have improved responses to therapeutic targets. CtDNA may play an important role in surveillance after tumor resection and may be used to reveal mechanisms of treatment resistance. The clinical utility of ctDNA has yet to be established. Significantly more research is needed to understand the utility ctDNA has in clinical practice.

**Abstract:**

CtDNA is emerging as a non-invasive clinical detection method for several cancers, including genitourinary (GU) cancers such as prostate cancer, bladder cancer, and renal cell carcinoma (RCC). CtDNA assays have shown promise in early detection of GU cancers, providing prognostic information, assessing real-time treatment response, and detecting residual disease and relapse. The ease of obtaining a “liquid biopsy” from blood or urine in GU cancers enhances its potential to be used as a biomarker. Interrogating these “liquid biopsies” for ctDNA can then be used to detect common cancer mutations, novel genomic alterations, or epigenetic modifications. CtDNA has undergone investigation in numerous clinical trials, which could address clinical needs in GU cancers, for instance, earlier detection in RCC, therapeutic response prediction in castration-resistant prostate cancer, and monitoring for recurrence in bladder cancers. The utilization of liquid biopsy for ctDNA analysis provides a promising method of advancing precision medicine within the field of GU cancers.

## 1. Introduction

CtDNA is released through tumor cell apoptosis and necrosis with an estimated half-life of 16 min to 2.5 h [1]. CtDNA release is tumor-dependent, with factors such as size, metastasis, and stage impacting the amount of ctDNA found in blood [2,3]. As the genetic aberrations of a tumor evolve over time, as a result of therapy or with disease recurrence or progression, ctDNA represents an opportunity for real-time molecular profiling that would otherwise require frequent, invasive biopsies [4]. Advancements in methods of ctDNA detection, such as PCR, next-generation sequencing (NGS), and epigenetic targeted immunoprecipitation approaches, have improved practical feasibility and cost-effectiveness [5]. To date, blood-based specimens are the most studied liquid biopsy method. However, there is growing interest in samples obtained from non-blood samples, such as urine samples. Urine ctDNA sensitivity may be higher, particularly in cancers with direct contact with the urine, such as in several GU cancers [6]. As the field continues to advance, further refinement and standardization of methods of detection and sample collection will be essential to clinical validation of ctDNA.

**Figure 1:**
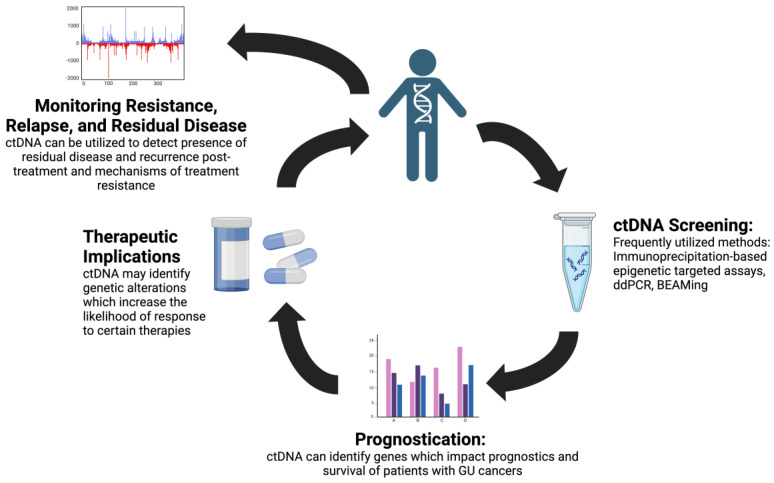
ctDNA utility in genitourinary cancers. CtDNA may have a role in screening for genitourinary cancers, prognostic implications, an ability to predict which patients may show improved response to targeted therapies, and an ability to monitor resistance, relapse, and residual disease. Figure 1 was created using BioRender.com (accessed on 18 June 2024).

Across various cancer types, ctDNA has emerged as a valuable tool for cancer screening, guiding treatment options with targeted mutation profiles, treatment response monitoring, and detection of residual disease [2]. ctDNA has the potential to improve the detection of GU cancers, which are often characterized by late presentation. As clinical trials increasingly incorporate genetic markers that stratify patients into treatment groups, ctDNA may be a valuable tool to risk-stratify patients for optimal therapeutic response. These applications of ctDNA hold promise to improve patient care by addressing critical gaps in GU cancer management. The points of intervention in which ctDNA may improve clinical care, including ctDNA-based cancer screening, prognostication, therapeutic response prediction, and cancer surveillance of relapse and residual disease, are demonstrated in Figure 1. Growing evidence supports the use of ctDNA in prostate and bladder cancer and RCC, while the study of ctDNA in adrenal, testicular, and penile cancer is less extensive, likely due to the lower incidence rates of these cancers. In this review, we discuss methods for ctDNA analysis, targets of ctDNA assays, the clinical significance of these assays, the prognostic value of ctDNA assays, and the treatment implications of these assays in GU tumors.

## 2. Detection Targets and Methods

### 2.1. Digital PCR and BEAMing

PCR methods, including droplet digital PCR (ddPCR) and BEAMing (beads, emulsion, amplification, and magnetics), are highly sensitive methods of detecting pre-targeted mutant alleles [5]. DdPCR is a highly sensitive test, enabling it to detect rare oncogenic mutations [7]. DdPCR has been used to detect plasma ctDNA in adult solid tumors with an 85.9% sensitivity rate and high accuracy when validated with tumor samples (>99% positive and negative percent agreements) and with some alleles detected at 0.001% [5,7]. BEAMing allows for similarly high sensitivity, detecting up to 0.01% of genomic material, by creating many individual DNA reactions with continuous amplification, followed by flow cytometry to assess for DNA variation [8,9]. DdPCR and BEAMing methods have been shown to have comparable cancer detection rates and high rates of ctDNA gene agreement between the two methods [9]. Several studies have developed GU tumor-specific gene panels with high sensitivity and specificity utilizing these PCR-based methods.

### 2.2. Ability to Detect Novel Genomic Alterations: Next-Generation Sequencing Methods

NGS methods enable the detection of untargeted genetic alterations and larger-scale targeted approaches. NGS methods such as TEC-Seq allow ultrasensitive evaluation through deep sequencing (~30,000×) of DNA fragments and have been used to detect cancers including colon, breast, and lung with high sensitivity [10]. Targeted panel ctDNA detection approaches retain high sensitivity with low costs and thus have been the methods relied upon for clinically available liquid-based biopsy tests, including Guardant 360 and Foundation Liquid CDx [5,11]. However, non-targeted NGS methods can be very costly depending on the depth of sequencing and are limited by estimated multi-week turnaround times, a limitation to broad clinical implementation [12]. However, García et al. found plasma ctDNA NGS testing accelerated time to treatment in lung cancer patients, finding patients with ctDNA testing prior to diagnosis had a median time to treatment of 39 days in comparison to 62 days for the reference group [13]. Whole-genome sequencing (WGS) and whole-exome sequencing (WES) allow for the detection of novel mutations and a more complete genomic picture than PCR [11]. However, WGS and WES are often more expensive, less sensitive, and require high sample volumes, limiting their use when ctDNA fraction is low [11].

### 2.3. Detection of Epigenetic Modifications: Targeting Histone Modification and Regulatory Elements

The focus of ctDNA has primarily been on targeting genomic alterations. However, targeting epigenomic features such as DNA methylation, nucleosome positioning, DNA fragmentation, and histone modifications allows for the detection of tumor gene regulation patterns [14]. Immunoprecipitation-based assays targeting histone promoter and enhancer modifications and methylated DNA in plasma samples have yielded important diagnostic markers and drug targets for different cancers [14]. Detection of methylation patterns may be a promising tool for utilizing ctDNA as a screening method that can detect early-stage disease. While somatic mutations accumulate slowly and thus may not be detectable in early-stage disease, cancer-specific epigenetic alterations can be found in early-stage neoplasms, suggesting that ctDNA assays which detect epigenetic modifications may be useful in the setting of low tumor burden [15]. Detection of ctDNA methylated regions successfully identified over 50 cancers in both early and late stages [16]. However, ctDNA methylation assays are limited by potentially reduced sensitivity due to epigenetic alteration variability and ambiguous or difficult-to-interpret assay results [17,18]. The detection of histone modification has the benefit of identifying the tumor of origin, which is essential to blood-based cancer detection methods [15,19]. Histone modification may help reveal the origin of metastatic cancer via the detection of cancer-specific methylation signatures, with one study reporting the correct identification of 96% of colorectal metastasis to the lung and 94% of colorectal cancer metastasis to the lung [11,20]. Table 1 compares the methodology, sensitivity, studied applications, and sensitivities of the different ctDNA detection methods discussed. 

### 2.4. Non-Invasive Detection of Evolving Genetic Profiles

As oncogenesis stems from the accumulation of genetic mutations and clonal selection, the genomic profile of GU neoplasms evolves with progression and metastasis. CtDNA presents a practical advantage over traditional tumor biopsy by enabling the detection of an evolving, heterogeneous, and metastatic genomic profile. The ease of liquid biopsy from plasma or urine makes real-time testing more feasible. This also eliminates the need to obtain biopsies from challenging primary and metastatic locations, such as in prostate cancer, where 90% of metastasis is to bone [21]. Due to tumor heterogeneity, tumor biopsy can lead to incomplete detection of genetic mutations, as reported by Phillimore et al., who found nearly 69% of all somatic mutations varied depending on the biopsy site [22]. Complementary use of ctDNA may reveal a more complete genetic picture by enabling the detection of tumor heterogeneity. Obtaining a more complete genetic profile may more accurately identify patients who may respond to certain targeted therapies and expand therapeutic options for patients.

Distant metastasis is a significant cause of cancer-related deaths, indicating the need for improved detection methods. Metastases can acquire unique genomics characteristics that differ from the original tumor and are distinctive from other metastatic sites [22]. These features prevent traditional tissue biopsies from accurately reflecting cancer genomics and present an opportunity for liquid biopsy biomarkers to improve prognostication of metastatic disease [23]. Clinton et al. report a 23% discordance between primary tumors and metastatic sites in urothelial cancers, creating barriers to precision medicine when relying solely on traditional, direct tissue-based methods [24]. Herberts et al. report that in a same-day analysis of metastatic tumor biopsy and ctDNA samples, when single clone expansion was identified in a metastatic tumor sample, plasma ctDNA detection revealed these to be subclonal populations in twelve of thirteen patients with prostate cancer [25]. Biopsied metastatic tissue contribution represented 19% of total ctDNA, with 97% of mutations in metastatic tissue samples detected by plasma ctDNA [25]. Wyatt et al. report that in patients with ctDNA fractions comprising over 2% of total circulating DNA, ctDNA identified all somatic mutations in matched metastatic biopsy in prostate cancer [26]. Actionable prostate cancer genetic alterations in the *AR*, *WNT*, and *PI3K* pathways were detected in ctDNA but not in metastatic biopsy, suggesting ctDNA may provide a more comprehensive genetic analysis than a single biopsy can offer [26].

## 3. Clinical Significance

### 3.1. Prostate Cancer

#### 3.1.1. Prostate Cancer Detection

Effective cancer screening and early detection are essential to reduce cancer-related mortality on a population level. Prostate-specific antigen (PSA) screening guidelines recommend a 4.0 ng/mL cutoff value, which yields a sensitivity and specificity of 21% and 91%, respectively [27]. PSA screening can lead to overdiagnosis and treatment, with studies estimating that 15% of men screened every 2 to 4 years will have a false positive test before ten years of screening [28]. Diagnostic testing via transrectal ultrasound-guided biopsy is often unnecessarily performed, which can lead to complications such as hematuria, as well as adverse effects such as infection, including sepsis [27,29,30]. Overdiagnosis and treatment of prostate cancer can cause significant morbidity, as well as economic and psychological harm. Although multi-parametric magnetic resonance imaging has been demonstrated to have a higher sensitivity (93%), its reported lower specificity (41%) signifies the importance of a non-invasive tool that can accurately determine clinically significant disease [5,30]. A direct, non-invasive screening method could complement PSA screening to detect prostate cancer with high sensitivity and improved specificity. Current large-scale endeavors to create a highly sensitive ctDNA screening tool include the Circulating Cell-Free Genome Atlas study, which has recruited over 15,000 participants to track biospecimens over five years through NGS [31].

Although ctDNA shows promise as a screening tool in other cancers, low levels of ctDNA have been found in localized prostate cancer, in contrast to the high ctDNA burden in mCRPC [32]. Low-level detectable ctDNA in early-stage prostate cancer challenges its utility as a screening method. For instance, Hennigan et al. failed to detect ctDNA in any patients with localized prostate cancer using ultra-low-pass WGS and targeted resequencing [32]. However, several alternative methods of ctDNA detection, including epigenetic biomarkers and fragementome analysis, have shown high sensitivity for prostate cancer detection. Epigenetic changes that have been studied as biomarkers of early prostate cancer include focal hypermethylation of CpG islands, long-range hypomethylation, and partially methylated domains [33,34]. Methylation panels targeting CpG islands have been used to detect over 50 cancer types with assay sensitivity dependent on the cancer stage [16]. Klein et al. investigated an early detection, multi-cancer methylation assay in a cohort of 4077 subjects, including patients with prostate, urothelial, and kidney cancer diagnoses [35]. The assay demonstrated an overall specificity of 99.5% and sensitivity of 51.5%, which, if implemented, would result in an estimated decrease of 104 deaths per 100,000 individuals screened [35,36]. However, in subgroup analysis, prostate cancer was detected with a sensitivity of only 11.2%, with the indolent early course and low prostate cancer tumor shedding as the probable explanation [35].

Studies utilizing assays targeting methylation patterns specific to prostate cancer and urinary ctDNA methods have shown improved sensitivity and specificity. Ellinger et al. found that ctDNA-detected CpG island hypermethylation differentiated prostate cancer and benign prostate hyperplasia (BPH) with a specificity of 92% and sensitivity of between 42 and 47% [37]. Brikun et al. employed 24 methylation-specific quantitative markers from 19 CpG islands associated with prostate cancer in urine samples to detect early prostate cancer. A combination of 6 of the 19 methylation markers detected prostate cancer with 71% specificity and 94% sensitivity from first-void urine [38]. Haldrup et al. detected prostate cancer via ctDNA detection of *STGGALNAC3*, *ZNG660*, *CCDC181*, and *HAPLN3* promoter methylation [39]. *ST6GALNAC3* identification resulted in a receiver operating characteristic (ROC) of 0.917–0.995, indicating a high ability to discriminate between those with and without prostate cancer [39]. A ctDNA hypermethylation assay detecting the *STGGALNAC3*, *CCDC181*, and *HAPLN3* genes was a 100% specific and 67% sensitive test [39]. Constâncio et al. created a plasma ctDNA assay that detected methylation levels of *FOXA1*, *RARβ2*, and *RASSF*, which identified prostate cancer with 64% sensitivity and 70% specificity from samples of 102 lung cancer, 121 prostate cancer, 100 colorectal patients, and 136 healthy subjects [40]. A predictive model using PSA, quantified ctDNA levels, and ctDNA-detected *GADD45* methylation in 34 prostate cancer and 48 healthy subjects yielded a specificity of 87.5%, a sensitivity of 94.1%, and a ROC of 0.937 [41]. O’Reilly et al. used a targeted six-gene hypermethylation panel in urine samples to detect aggressive prostate cancer with an area under the ROC curve (AUC) of 0.64 [42]. They further distinguished aggressive high-grade prostate cancer (Gleason score ≥ 8) with an AUC of 0.86 and high-risk aggressive prostate cancer (as defined by Cancer of the Prostate Risk Assessment) with an AUC of 0.83 [42]. Methylation of *GSTP1* occurs in 90% of prostate cancers, with potential for detection in serum, plasma, urine, and ejaculate [43]. A meta-analysis including 16 studies detecting *GSTP1* methylation in plasma, serum, and urine samples compared to healthy controls reported a specificity of 0.89 (95% CI, 0.80–0.95) and sensitivity of 0.52 (95% CI, 0.40–0.64) [44]. Fragmentome analysis of the first coding exon in ctDNA sequencing panels detected prostate cancer from a cohort of 198 breast, lung, prostate, and non-cancer subjects with an accuracy of 76.3% in the validation cohort [45]. These epigenetic alterations captured via ctDNA may hold promise to improve the early detection of prostate cancer.

#### 3.1.2. Prostate Cancer Prognostics

The ability of blood- and urine-based assays to detect primary and metastatic tumor profiles could facilitate tumor risk stratification. Current tools for identifying genetic alterations largely focus on tumor samples, such as the Decipher test, a 22-gene test utilizing tumor biopsy, which has been validated as a prognostic tool for predicting metastasis and risk classification following prostatectomy [46,47]. Deep WGS of ctDNA characterized genomic alterations in aggressive prostate cancer, aligning with findings from tumor-based genetic studies: *TP53* (73% of patients), *PTEN* (48% of patients), *RB1* (18% of patients), *ETS* fusion (49% of patients), and DNA repair defects (27% of patients) [25]. Integrating blood-based ctDNA assays would expand the ease of delivering personalized targeted therapy.

Several studies have investigated the feasibility of ctDNA as a predictive tool in prostate cancer, particularly in metastatic castration-resistant prostate cancer (mCRPC). Clinically actionable genetic alterations are estimated to be found in 89% of patients with mCRPC [48]. The detection of these genes via ctDNA may confer specific prognostic capabilities. Van Dessel et al. performed WGS on tumor biopsy and matched blood samples reporting microsatellite instability (MSI), homologous recombination deficiency, CDK12 mutations, and androgen receptor (AR) alterations, which could stratify patients into clusters to guide clinical decision-making [49]. The FDA-approved Guardant360 assay, a clinically available tool that characterizes 72 somatic cancer-associated genes via ctDNA, found that 94% of a 514 patient cohort with mCRPC had detectable ctDNA alterations [50]. Distinct ctDNA alterations, including *AR* alterations, as well as *MYC* and *BRAF* amplifications, were associated with poorer clinical outcomes and therapeutic responses [50]. These findings parallel those reported in a broader cohort of 3129 patients [51]. The FoundationOne Liquid CDx biopsy, an NGS method that detects over 300 genetic alterations from peripheral whole blood, also detected ctDNA from 94% of patients with advanced prostate cancer [51]. *AR* alterations were detected in 42% of the cohort, while *BRCA1/2* alterations were reported in 8.8% of the cohort [51]. These genetic alterations have clinically actionable targets, which may improve personalized treatment options.

CtDNA can predict treatment response to next-line therapy in mCRPC. Annala et al. employed targeted exon sequencing to risk-stratify 201 patients with mCRPC randomized to abiraterone or enzalutamide treatment [52]. Quantification of high ctDNA (30–100%) predicted a mean overall survival (OS) of 10.1 months with a hazard ratio (HR) of 12.92 (*p* < 0.001) [52]. By comparison, PSA > 40 ng/mL was associated with a median survival of 16.7 months and HR of 3.87 (*p* < 0.001) [52]. CtDNA identification of *BRCA2* and *ATM* truncating mutations were associated with a median time to progression (TTP) of 1.8 months and univariate HR of 6.14 (*p* < 0.001), compared to an all-patient TTP of 7.5 months [52]. *AR* defects also predicted shorter TTP, with increasing copy number (CN) variants equating to shorter survival times [52]. AR structural rearrangements were associated with primary resistance, likely accounting for shorter TTP [52]. Patients with ctDNA detection ≥ 5% had significantly shorter OS and TTP, with a mean time from the start of the first-line treatment to progression of 7.8 months, compared to 19.1 months for patients with <5% detectable ctDNA (*p* < 0.001) [53]. Romanel et al. found that 45% of patients in a cohort of 97 patients with mCRPC had *T878A* or *L703H* AR changes before initiating abiraterone therapy, which was associated with poorer outcomes [54]. Chi et al. report similar predictors of shorter TTP in patients with mCRPC treated with abiraterone or enzalutamide, including baseline ctDNA > 2% (HR 1.80, *p* = 0.005), *TP53* (HR 2.84, *p* < 0.001), *RB1* (HR 1.96, *p* = 0.002), *AR* (HR 2.04, *p* < 0.001), and DNA repair alterations (HR 4.13, *p* < 0.001) [55]. CtDNA may serve as a valuable adjunctive tool to tumor tissue biopsy in risk-stratifying patients by enabling the detection of genetic alterations with prognostic significance.

#### 3.1.3. Prostate Cancer Therapeutic Implications

##### Androgen Receptor Alterations

Effective detection of genetic biomarkers is pivotal for optimizing gene-based therapeutic strategies and improving clinical decision-making [56]. CtDNA stands out as a biomarker capable of non-invasively detecting clinically actionable genetic alterations. CtDNA-based assays have gained FDA approval for guiding therapy decisions in non-GU cancers, and ongoing research in GU cancers demonstrates the ability of ctDNA to identify targetable alterations, including those in the AR signaling pathway [57].

Although androgen receptor signaling inhibitors (ARSIs) such as enzalutamide and abiraterone exhibit survival benefits in mCRPC, primary resistance is observed in 40% of cases, and secondary resistance is eventually evident in nearly all cases [58,59,60]. Enzalutamide and abiraterone target the ligand binding domain (LBD) and downstream AR signaling [61,62]. CtDNA can detect alterations in LBD affinity or structure and AR amplifications, which may play a role in predicting therapeutic responses [53,63]. Herberts et al. report that 95% of ctDNA samples from patients with CRPC revealed AR alterations, including LBD missense mutations (18%), AR amplifications (48%), and LBD structural alterations (7%) [25]. AR copy number gain or LBD alterations contribute to therapeutic resistance to these agents, underscoring the role ctDNA has in predicting treatment responsiveness [64].

Comprehensive ctDNA analysis has identified specific prognostic markers of ARSI therapy response, with significant attention garnered to the AR splice variant 7 (AR-V7), which codes for a truncated LBD. CtDNA-detected AR-V7 alterations can predict response rates to enzalutamide and abiraterone. Patients with AR-V7 mutations treated with enzalutamide or abiraterone exhibited lower PSA response rates compared to patients without (ENZ: 0 vs. 53%, ABI: 0% vs. 68%, *p* = 0.004), shorter PFS (ENZ: 1.4 months vs. 6.0 months, ABI: 1.3 months vs. not reached, *p* < 0.001), and shorter OS (ENZ: 5.5 months vs. not reached, *p* = 0.002; ABI: 10.6 months vs. not reached, *p* = 0.006) [58]. Armstrong et al. reported that ctDNA detection of AR-V7 was associated with shorter PFS (HR 2.4, 95% CI: 1.1–5.1 *p* = 0.02) and OS (HR: 3.5) with abiraterone or enzalutamide in patients with mCRPC [65]. Detecting AR-V7 by plasma DNA could provide important insights into anti-androgen therapy response [65].

Other AR alterations detected by ctDNA may also predict ARSI response. Conteduca et al. found AR gain was associated with worse overall survival (HR of 3.98, *p* < 0.001) and PFS (HR 2.18, *p* = 0.03) with abiraterone and enzalutamide treatment [66]. CtDNA-detected AR gain varied with the prior line of therapy; 14% of chemotherapy-naïve patients had ctDNA-detected AR gain compared to 34% of patients treated with docetaxel [66]. In patients treated with enzalutamide and no prior chemotherapy, AR gain was associated with worse PSA PFS (HR 4.33, *p* < 0.001), radiographic PFS (HR 8.016, *p* < 0.001), and OS (HR 11.08, *p* = 0.004) [66]. Plasma ctDNA analysis revealed AR status before initiating enzalutamide or abiraterone was an independent predictor of treatment outcome [66]. Da Laere et al. found AR changes in 25% of patients with mCRPC [67]. Fourteen patients had AR splice variants, which predicted worse PFS to second-line androgen therapy (HR 4.53, 95% CI, 1.424–14.41; *p* = 0.0105) [67]. CtDNA may better inform which patients may benefit from ARSIs. Prospective randomized trials investigating AR status are necessary to validate these findings.

##### DNA Repair Defects and PARP Inhibitor Response

CtDNA can detect homologous recombination repair (HRR) defects such as *BRCA2*, which has garnered attention for its enriched response to PARP inhibitors [5,68]. Germline DNA repair defects, including *BRCA2*, *ATM*, *CHEK2*, *BRCA1*, *RAD51D*, and *PALB2*, have been identified in 11.8% of patients with metastatic prostate cancer and 2.7% of patients with localized disease [69]. PARP inhibitors demonstrate improved overall response rate (ORR) in patients with germline and somatic *BRCA* alterations [70]. In the PROfound trial, a crossover randomized phase III trial, patients with mCRPC with *BRCA1*, *BRCA2*, and *ATM* tumor alterations showed improved survival when treated first-line with olaparib compared to enzalutamide or abiraterone (19.1 months vs. 14.7 months, HR = 0.69; *p* = 0.02) [71]. This trial confirmed the results from TOPARP-A, a phase II trial of olaparib in patients with metastatic prostate cancer, which demonstrated high response rates to olaparib in patients with defective DNA damage repair (DDR) [72]. The DDR-dependent olaparib response underscores the potential of ctDNA as a biomarker of tumor genetic alterations in a subset of patients.

CtDNA-detected *BRCA* and *ATM* genes exhibited an 81% positive percentage agreement and 92% negative percent agreement with tumor tissue biopsy [73]. Goodall et al. studied ctDNA WES in TOPARP-A, where ctDNA was identified in 93% of patients and captured all *BRCA1*, *BRCA2*, and *ATM* alterations identified in tumor biopsies, along with newly identified mutations associated with disease progression [74]. Quantified ctDNA levels declined in olaparib-responding patients, which were associated with radiological PFS (HR 0.41, *p* = 0.009 for >50% fall of baseline ctDNA). CtDNA decline at eight weeks correlated to longer rPFS and OS [74]. Five of the six subjects bearing somatic homologous recombination deficiency (HRD) mutations at baseline responded to treatment and had a corresponding decrease in ctDNA to <5% in HRD genes [74]. CtDNA WES identified several resistance mechanisms to olaparib therapy, revealing the reversion of DDR mutations upon progression in olaparib therapy [74]. Goodall et al. and Quigley et al. both report ctDNA-detected *BRCA2* reversion mutations that were not present before PARP therapy, implying DDR restoration as a mechanism of PARP resistance [74,75].

The FoundationOne Liquid CDx was FDA-approved for use in prostate cancer specifically to identify patients with *BRCA1/BRCA2* mutations and qualify them as eligible for rucaparib treatment [76,77]. The FoundationOne liquid biopsy claims a reproducibility of 99.59%, a positive percent agreement of 96.3%, and a negative percent agreement of >99.9% [77]. FDA approval of a liquid biopsy method for patient selection to PARP inhibitors represents a milestone for clinical implementation of ctDNA.

##### PTEN-PI3K-AKT Pathway Detection and Ipatasertib Response

Treatment with ipatasertib, an AKT inhibitor, and abiraterone showed improved radiographical PFS compared to abiraterone plus placebo in patients with tumor *PTEN*-loss (18.5 months vs. 16.5 months, HR 0.77; 95% CI 0.61–0.98; *p* = 0.034) [78]. This presents an opportunity to employ ctDNA to detect *PTEN* loss [79]. Dong et al. used an NGS-based ctDNA assay and low pass-WGS to detect *PTEN* loss in mCRPC patients [80]. A 100% agreement was reported for *PTEN* loss between matched plasma and tumor samples, while the overall assay had an 86.7% concordance rate [80]. The assay exhibited 80% sensitivity, 100% specificity, and 100% PPV for *PTEN* loss [80]. Wyatt et al. report an 88.9% concordance between tumor and tissue samples for clinically actionable genes, including *PTEN* [26]. Utilizing ctDNA to detect PTEN-PI3K-AKT pathway deregulation represents an opportunity to guide PTEN-PI3K-AKT-targeting treatments, such as ipatasertib.

##### Microsatellite Instability and Immune Checkpoint Inhibitor Response

Pembrolizumab is the only immune checkpoint inhibitor (ICI) currently FDA-approved for use in prostate cancer, and this approval is limited to use in a subset of patients with high MSI, mismatch repair deficiency (dMMR), or a high number of mutations per mega-base, termed tumor mutational burden (TMB) [81]. dMMR-altered cancer represents a genomic signature with a significantly higher mutational rate than tumors with proficient MMR. Neoantigens resulting from dMMR are hypothesized to promote an enhanced immune response and thus improve responsiveness to ICIs [25,82,83,84]. A total of 54.5% of patients with MSI/dMMR-positive prostate cancer were found to respond to anti-PD-1/PD-L1 treatment, with greater than 50% PSA decline [85]. Although found in only 3.1% of patients, MSI/dMMR stands out for its clinical usefulness as a biomarker with a targeted therapy [85]. In a study of two hundred and thirty-three patients with MSI/dMMR-positive cancers, including six patients with prostate cancer, five patients with adrenocortical cancer, five patients with urothelial cancer, three patients with renal cancer, and one patient with testicular cancer, 34.3% of the overall population responded to pembrolizumab [82]. Among patients that responded, 86.9% had a duration of 12 months or longer [82]. CtDNA can provide real-time complementary information to detect dMMR alterations and identify a subset of patients with prostate cancer that may respond to pembrolizumab [25]. The genetic targets detected by ctDNA, corresponding drug targets, and treatment agents for prostate cancer are displayed in Table 2. 

#### 3.1.4. Prostate Cancer Resistance, Relapse, and Residual Disease

Extensive research has been dedicated to elucidating AR evolution and subsequent treatment resistance during ARSI treatment via ctDNA [53]. Herberts et al. employed WGS of ctDNA to track mCRPC evolution and clonal population development [25]. A mean of 2.9 subclonal populations per patient was reported, which was significant to clinical prognostication as tumoral heterogeneity can contribute to treatment failure [25,86]. Of patients treated with an ARSI, 62% had temporal changes in the AR, including gene body and enhancer amplifications, LBD switches, and CN changes [25]. In a crossover trial of 202 patients, ctDNA identified pre- and post-treatment AR copy number alterations, AR enhancer amplification, and structural AR changes [53]. Most somatic driver mutations detected in pre-treatment persisted in post-treatment samples; however, 36% of patients had tumor population shifts [53]. Annala et al. report that 53% of patients had ARSI-induced ctDNA changes, 86% of which were AR locus changes [53]. All patients with >90% PSA decline and >10% ctDNA detection had tumor population shifts, indicating treatment-induced gene alteration pressures [53]. These studies demonstrate the ability of ctDNA to track ARSI resistance in real time.

More specific mechanisms of ARSI resistance have been revealed via longitudinal ctDNA analysis. Annala et al. found a notable increase in AR *L702H* mutations after abiraterone, enzalutamide, and glucocorticoid treatment [53]. Wyatt et al. similarly reported new or increased L702H mutations with enzalutamide treatment, whereas *W742LC* mutations regressed during treatment [87]. Post-bicalutamide treatment, shifts from AR 742 to 743 mutations were found, aligning with prior evidence of this resistance mechanism [53,88]. During treatment, the average AR copy number increased from 7.8 to 10.1 to 12.3 copies in sequential collection; a higher AR copy number was associated with a shorter OS [53]. Whole-genome analysis revealed recurrent changes in the WNT pathway, PI3K pathway, and cell cycle gene alterations [53]. Sonpavde et al. conducted serial ctDNA profiling, detecting AR variants *L702H*, *T878A*, *H875Y*, *W742C*, and *F877L*, known to contribute to ARSI resistance, in addition to new *AR*, *MET*, *PIK3CA*, *BRAF*, and *MYC* alterations [50]. Wyatt et al. detected AR mutations in 48% of patients pre-enzalutamide treatment and 60% of patients post-progression in a cohort of 65 patients with mCRPC [87]. During enzalutamide treatment, *MET* gain, *RB1* loss, and multiple AR mutations, including AR gain or amplification, were associated with HRs of 4.53, 4.46, 3.94, and 2.92, respectively [87]. AR amplification was exclusively seen post-ARSI treatment [87].

CtDNA can detect disease progression, in some cases, before clinical evidence is apparent. Romanel et al. found a ctDNA-detected AR point mutation conferring resistance several months before clinically observable progression [54]. Tolmeijer et al. found persistent ctDNA was associated with shorter PFS and OS after four weeks of ARSI treatment [89]. Conteduca et al. reported that ctDNA rise during abiraterone treatment was associated with an odds ratio of 15.8 for early radiographic progression (*p* = 0.0002) and an associated OR of 6.0 for PSA rise [90]. Jayaram et al. conducted a phase II trial comparing ctDNA pre- and post-abiraterone treatment, finding that pre-treatment AR gain was consistently present at progression, and ligand receptor AR mutations were solely present at progression [91]. Patients who achieved undetectable ctDNA levels while receiving abiraterone showed survival like those with undetectable pre-treatment ctDNA [91]. Wyatt et al. detected ctDNA in 28% of patients during enzalutamide treatment, while at progression, 70% of patients had detectable ctDNA [87].

CtDNA methylation patterns exhibit alterations in response to successive lines of therapy. Silva et al. reported temporal changes in methylated genes associated with taxane and AR agent treatment [92]. Silva et al. found recurrent prostate cancer hypomethylation patterns, such as *EPN1* and *CPEB4*, which are known to promote prostate cancer progression, but reported that most temporal methylation patterns were patient-specific [92,93]. Mahon et al. studied mGSTP1 as an epigenetic biomarker to track disease progression. After docetaxel treatment, undetectable mGSTP1 was associated with a longer TTP and OS [94]. CtDNA monitoring can offer real-time detection of treatment resistance and disease progression in prostate cancer via various mechanisms.

### 3.2. Bladder Cancer

#### 3.2.1. Bladder Cancer Detection

CtDNA screening has the potential to reduce morbidity resulting from standard-of-care bladder cancer diagnostic procedures, such as cystoscopy, while also improving outcomes through earlier-stage diagnosis. CtDNA has been found in the urine and plasma of patients with non-invasive bladder cancer, indicating ctDNA as a screening tool for patients with disease that does not have basement membrane invasion [95]. CtDNA can be detected in 75–90% of urothelial cancers, placing it among the cancers with the highest ctDNA levels [96]. Klein et al. identified bladder cancer via epigenetic methylation ctDNA assays with stage-specific rates of 33.3%, 9.1%, 75%, and 100% for stages I, II, III, and IV, respectively [35]. Many studies have detected bladder cancer with greater efficacy using urinary ctDNA, likely attributed to the direct contact of the tumor with urine.

Several methods of urinary ctDNA bladder cancer detection have been studied, including targeting the widely present *TERT* mutation and epigenetic modifications. Urinary *TERT* promoter mutations have been identified with a sensitivity of 55.56% and specificity of 100% [97]. A high-throughput sequencing method detecting recurring literature-reported bladder cancer mutations via urinary ctDNA reported an 84% sensitivity and a 96–100% specificity [98]. The *TERT* and *PLEKHS1* promoters were most commonly mutated, with incidences of 75% and 46%, respectively [98]. In a cohort of 355 patients with either bladder cancer or non-malignant hematuria, *IQGAP3/BMP4* and *IQGAP3/FAM107A* urine PCR ratios yielded a sensitivity of 71.0%, specificity of 88.6%, and AUC of 0.862 [99]. Cheng et al. targeted urinary ctDNA methylation and copy number aberrations to detect BC with a sensitivity of 93.5% and specificity of 95.8% [100]. However, when stratified, low-grade non-muscle-invasive bladder cancer (NMIBC) was detected with a sensitivity of 84.2% [100]. Xu et al. reported urinary ctDNA-detected methylation of the *TERT* promoter and *ONECUT2* CpG sites identified upper tract urinary carcinoma with 94.0% sensitivity, 93.1% specificity, and an AUC of 0.961 in the validation dataset [101]. Zeng et al. used low-coverage WGS of urinary ctDNA to detect urothelial cancers with a sensitivity of 82.5%, specificity of 96.9%, and accuracy of 89.0% [102]. This accumulating body of evidence suggests urinary ctDNA could function as a sensitive tool for bladder cancer screening.

#### 3.2.2. Bladder Cancer Prognostics

CtDNA holds promise for enhancing prognostication in bladder cancer via quantification of genetic material and identification of specific alterations. Christensen et al. reported a hazard ratio of 29.1 (*p* = 0.001) for patients with localized advanced bladder cancer with detectable ctDNA compared to those without [103]. A total of 89% of patients with detectable plasma ctDNA developed recurrent disease, compared to 33% of patients without detectable ctDNA [104]. Shohdy et al. reported that serial ctDNA measurements could predict durable clinical responses in 90% of patients with urothelial carcinoma [105]. Specifically, lower variant allele frequency (VAF), the proportion of an allele within a sample, was predictive of a longer overall survival (HR: 0.31, 95% CI: 0.11–0.90, *p* = 0.03) [105]. At progression, 30% of patients were found to have a clinically actionable alteration, suggesting serial ctDNA measurements can help guide treatment decisions [105].

The identification of specific genetic aberrations also provides prognostic information. In advanced urothelial cancer patients, Grivas et al. and Shohdy et al. report similar incidences of ctDNA genomic alterations: *TP53* (found in 54.8–64% of patients), *PIK3A* (19–24.2%), *ARID1A* (22.6%), *ERBB2* (19–19.4%), *EGFR* (16.1–19%), *NF1* (13.7%), *RB1* (12.9%), *FGFR3* (11.3–19%), *BRAF* (10.5%), *BRAC1* (10.5%), and *RAF1* (8.9%) [105,106]. Some of these genetic aberrations yielded prognostic value, such as *BRCA1* and *RAF1*, which predicted a shorter OS and failure-free survival (FFS) [106]. Christensen et al. report urinary ctDNA detection of *FGFR3* and *PIK3CA* in NIMBC as prognostic to disease progression (*p* < 0.001) [104]. Monitoring *FGFR3* and *PIK3CA* showed efficacy in rapidly assessing disease progression [104]. In a metastatic bladder cancer profile, Vandekerkhove et al. found *TP53*, *RB1*, and *MSM2* to be altered in 95% of bladder cancer [107]. Puntoni et al. reported serum VEGF in the top quintile of a cohort of 99 patients with resected bladder tumors was an independent prognostic factor for OS (HR 2.7) and bladder cancer-specific survival (HR = 8.9) [108]. CtDNA-based detection of genetic alterations may better inform disease course and treatment options. In the future, utilizing broader gene panels may enhance the accuracy of prognostication of bladder cancer [104].

CtDNA and urinary ctDNA can also predict neoadjuvant and adjuvant therapy responses [109]. Christensen et al. found that ctDNA detection prior to neoadjuvant therapy was associated with subsequent lower response rates (*p* < 0.001), and detection of plasma and urinary DNA following neoadjuvant therapy was associated with lower response rates [109]. Combined urine and plasma DNA detection was able to predict treatment response and recurrence-free survival [109]. In an analysis of a randomized phase III trial of 581 patients with urothelial carcinoma being monitored for recurrent disease post-resection, ctDNA was detected in 37% of patients and was associated with poorer outcomes, likely as ctDNA served as a biomarker of molecular residual disease [110]. However, patients with detectable ctDNA demonstrated improved responses to atezolizumab therapy (hazard ratio = 0.58) compared to those without detectable ctDNA [110]. Thus, CtDNA identified a subset of patients who are both more likely to relapse and who demonstrate benefit from adjuvant therapy, having important implications for selecting which patients may benefit from specific treatment plans [110]. This highlights the role ctDNA could have in predicting neoadjuvant and adjuvant therapeutic response in urothelial carcinoma.

CtDNA can detect an evolving genomic landscape from multiple metastatic sites, which improves the ability to predict disease trajectory. Large-scale studies of tumor tissue (Ross et al. and Robertson et al.) and ctDNA (Agarwal et al., Grivas et al., and Shohdy et al.) found targetable mutations in urothelial cancer at similar rates, including *TP53* (48–64%), *PIK3CA* (14–24.2%), *FGFR3* (10–21%), and *ERBB2* (10–19.4%) [96,105,106,111,112]. Vandekerkhove et al. report that ctDNA and tissue biopsy displayed a concordance rate of 83.4%, in which ctDNA consistently recognized driver mutations [113]. Although serial ctDNA samples consistently identified 90% of mutations, significant heterogeneity existed between primary tumors compared to tumor samples taken at different time points, which is hypothesized to indicate clonal progression [113]. Barata et al. report that ctDNA analyzed by Guardant360 demonstrated a concordance rate of 16.4% with tumor samples analyzed via FoundationOne collected simultaneously in advanced urothelial cancer [114]. This suggests that ctDNA may be able to obtain a more complete genomic profile in metastatic bladder cancer. The ability of ctDNA to detect real-time genomic changes and a metastatic genetic profile would greatly improve bladder cancer prognostication.

#### 3.2.3. Bladder Cancer Therapeutic Implications

##### Immune-Modulating Therapies

Bladder cancer is one of the cancers with the highest TMB, which may lead to higher response rates to treatment with ICI, which ctDNA can be used to predict [115,116,117]. In a phase II trial of patients with locally advanced and metastatic urothelial carcinoma treated with atezolizumab, an anti-PD-L1 therapy, the median mutational load was 12.4/Mb in responders, compared to 6.4/b in non-responders (*p* < 0.0001) [118]. Necchi et al. found that PD-L1, TMB, and DDR in pre-therapy lesions in muscle-invasive bladder cancer (MIBC) were significantly different in patients with a complete response to pembrolizumab in comparison to those without [119]. Significant genetic changes and lower TMB post-therapy suggested possible mechanisms of pembrolizumab resistance [119]. Toripalimab, an anti-PD-1 therapy, has shown improved ORR and PFS in patients with metastatic urothelial cancer with detectable PD-L1 expression and higher TMB [120]. Early ctDNA response to toripalimab was associated with increased survival time [120]. Zang et al. report that ctDNA copy number abnormalities and cancer cell fraction could predict toripalimab responders versus non-responders with an accuracy of 90% [121]. These studies highlight the use of ctDNA to select a subset of patients with differential responses to anti-PD-L1 and anti-PD-1 antibody therapy. In contrast to the two-year disease-free survival (DFS) and OS in a phase II trial of patients with cisplatin-ineligible MIBC treated with two cycles of atezolizumab pre-radical cystoscopy, TMB was not associated with relapse-free survival (RFS) [122]. However, ctDNA quantification was highly predictive of treatment outcomes [122].

Mean VAF can also predict ICI responses. CtDNA was detected in 29/29 patients with urothelial carcinoma in a phase I/II trial of durvalumab, finding that mean VAF in 73 genes could predict treatment response [123]. VAF < 0 at six weeks of durvalumab treatment was associated with a reduction in tumor volume and a longer treatment duration (13 months vs. 7 months) [123]. Furthermore, mean VAF correlated with PFS (1.6 months dVAF ≥ 0 vs. 13.8 months dVAF < 0, HR, 0.21) and OS (HR, 0.001) with durvalumab treatment [123]. In contrast, in a phase 1B trial of preoperative ipilimumab plus nivolumab in stage III urothelial carcinoma, mean VAF did not differ between responders and non-responders [124]. However, a significant association was detected between the absence of pre-radical cystectomy (RC) plasma and urinary ctDNA in responders compared to non-responders (HR: 10.4, 95% CI: 2.9–37.5) [124]. Further research is needed to clarify the impact of ctDNA-detected VAF on ICI response as the studies conducted by Van Dorp et al. and Raja et al. are limited by small sample size.

##### FGFR Mutations

*FGFR3* mutations have an estimated prevalence in 72% of low-grade NMIBCs and 80% of low-grade upper tract urothelial cancers [125]. CtDNA may play a role in identifying *FGFR* alterations and predicting response to FGFR inhibitors such as rogaratinib, edafitinib, furtibatinib, and pemigatinib [104,126]. The FORT-1 phase II/III randomized trial of rogaratinib in locally advanced or metastatic *FGFR1/3* mRNA-positive urothelial carcinoma reported no response benefit of rogaratinib compared to chemotherapy [127]. However, post hoc analysis of patients bearing *FGFR3* alterations found higher ORR to rogaratinib (52.4%) compared to chemotherapy (26.7%) [127]. Although a retrospective analysis, the improved ORR reported with *FGFR3* reveals an opportunity to explore ctDNA as a treatment-selective biomarker.

Facchinetti et al. investigated ctDNA and tumor tissue genetics in 21 *FGFR3*-altered urothelial cancers treated with selective FGFR inhibitors [128]. CtDNA was used to track resistance mechanisms; of the 19 patients with progression, ctDNA detected activating *FGFR3* mutations at progression, including in the *FGFR3* tyrosine kinase domain, as well as off-target resistance mechanisms in the PI3K-mTOR pathway [128]. In the analysis of 32 patients with urothelial carcinoma bearing *FGFR3* mutations, 40% responded to edafitinib treatment [126]. CtDNA unveiled resistance mechanisms involving *TP53*, *AKT1*, and alternative *FGFR3* alterations [126]. Further research is warranted to investigate ctDNA for use in FGFR inhibitor treatment stratification and identification of resistance mechanisms.

##### DNA Damage and Nucleotide Excision Repair Alterations and Platinum Sensitivity

Alterations in DDR genes, such as *ATM*, *RBI*, and *FANCC*, and nucleotide excision repair genes, including *ERCC2*, can predict response to cisplatin-based therapies in bladder cancer [129]. Galsky et al. reported significantly improved cisplatin and ipilimumab response rates in patients with DDR alterations [130]. Christensen et al. reported that plasma *ERCC2* mutations were associated with a higher response to chemotherapy (*p* = 0.024) [103]. Vandekerkhove et al. report that ctDNA detection of *ERCC2* mutations was associated with increased cisplatin sensitivity and improved PFS [113].

Although a ctDNA approach to targeted therapy is promising, a multi-arm study of biomarker-targeted therapies in advanced urothelial carcinoma, including FGFR inhibitors in tumors with detected *FGFR* alterations, PARP inhibitors in tumors with dHRR, and TORC1/2 inhibitors in mTOR/PI3K tumor pathways alterations, did not report improved responses with corresponding ctDNA-detected pathway alterations [131]. No significant differences were found in response rates, PFS, or OS compared to monotherapy controls, raising questions about the efficacy of a biomarker-targeted approach [131]. However, the study was limited by a small sample size and a design that lacked the ability of direct group comparisons [131].

#### 3.2.4. Bladder Cancer Resistance, Recurrence, and Residual Disease Monitoring

Nearly 50% of patients with MIBC have disease recurrence post-radical cystectomy. Thus, a non-invasive biomarker to detect disease recurrence is urgently needed to reduce morbidity and mortality [132]. Clinically available assays, such as the Signatera MRD test, are ctDNA-based tests used for risk stratification and monitoring of treatment response in bladder cancer [117,133]. In the ABACUS trial, ctDNA was found to be prognostic at baseline, after atezolizumab therapy, and post-radical cystectomy, with a prevalence of 63%, 47%, and 14% at the respective time points [122]. Post-radical cystectomy, ctDNA was undetectable in patients who responded or had stable disease. In comparison, 83% of relapsed patients had detectable ctDNA [122]. Continuous ctDNA measurements demonstrated an RFS hazard ratio of 78 (*p* < 0.001) [122]. Carrasco et al. report that ctDNA-detected mutations in *TERT* and *ATM* were predictive of tumor progression before radical cystectomy, at four months post-radical cystectomy, and 12 months post-radical cystectomy (HR 6.774, HR 3.673, and HR 30.865, respectively; *p* < 0.05) [134]. Patients with metastatic relapse post-radical cystectomy had significantly higher detectable ctDNA compared to disease-free patients (*p* < 0.001), which was detectable at a median of 137 days, compared to clinical diagnosis with cystectomy at 275 days [135]. Christensen et al. used ultra-deep sequencing methods to detect recurrence in localized advanced disease during chemotherapy and post-cystectomy [103]. CtDNA identified metastatic relapse with 100% sensitivity and 98% specificity [103]. CtDNA was observable 96 days before radiographic imaging [103].

Urinary ctDNA also can be used to detect bladder cancer recurrence. Dudley et al. detected urinary ctDNA in 91% of patients with recurrent disease [98]. Urinary ctDNA detection was in advance of clinical detection by 2.7 months, enabling earlier clinical actions to be taken [98]. In 12 patients with recurrent or metastatic bladder cancer, significantly higher urinary ctDNA amounts were found in patients whose disease progressed to MIBC than patients with NMIBC recurrence (mean 1282 copies/mL vs. 31 copies/mL, respectively, *p* = 0.007) [95]. Urinary ctDNA was higher in patients who progressed to MIBC, with a range of 12–169 months before clinical diagnosis [95]. Urinary ctDNA monitoring of disease recurrence would not only improve the ease of disease tracking but also improve prognosis due to earlier identification [95].

### 3.3. Renal Cell Carcinoma

#### 3.3.1. Renal Cell Carcinoma Detection

RCC represents 90% of all kidney cancers, with an increasing incidence rate [136,137,138]. Often, subtle signs and symptoms contribute to delayed diagnosis of RCC, and approximately one-third of patients with kidney cancer present with metastasis [138,139,140]. A reliable, non-invasive biomarker to detect RCC would vastly improve patient care. RCC has been reported as a cancer with one of the lowest amounts of detectable ctDNA in the serum, presenting challenges to ctDNA assays, particularly in early-stage disease [141]. However, targeting ctDNA epigenetic methylation patterns has been leveraged as a detection method that may recognize early-stage genomic changes [15].

Studies have detected RCC with high sensitivity and specificity by utilizing ctDNA methylation panels targeting commonly altered RCC genes, including *VHL*, *RASSF1A*, *P16*, *P14*, *RARB*, *TIMP3*, *GSTP1*, and *APC* [15,142]. A cell-free methylated DNA immunoprecipitation and high-throughput sequencing assay (cfMeDIP-seq) reported an AUC of 0.99 by detecting 300 distinct methylation areas in plasma samples from 69 patients with RCC and 13 healthy controls [140]. When urine samples were investigated using the cfMeDIP-Seq protocol, the AUC was reported as 0.86 [140]. Outeiro-Pinho et al. found methylation at the 5p promoter region (miR-30a0-5p^me^) identified ccRCC with an 83% sensitivity and 67% specificity [143]. Lin et al. detected *PCDHI7* methylation in 142 ccRCC and 34 healthy controls with a sensitivity of 57.7% and specificity of 100% [144]. Skrypkina et al. investigated methylation of six tumor suppressor genes in 27 RCC patients and 15 healthy subjects, finding *LRRC3B* detection yielded a sensitivity of 74.1% and specificity of 66.7%; *RASSF1* detection was a 62.9% sensitive and 93.3% specific test; *FHIT* detection was a 55.6% sensitive and 100% specific test; and *APC* detection was a 51.9% sensitive and 93.3% specific test [145]. Utilizing methylation detection across multiple genes enabled higher detection rates. For instance, the detection of *RASSF1* or *RHIT* or *APC* was a 92.3% sensitive and 86.7% specific test in RCC, while detecting *RASSF1* or *FHIT* was 77.8% sensitive and 93.3% specific [145]. Hoque et al. reported promoter methylation in urine or serum of RCC patients generated a 94% sensitivity and high specificity [146]. The emerging evidence supporting the utilization of ctDNA methylation detection in RCC is promising, but independent validation efforts are imperative to clinical use.

In addition to epigenetic targeted assays, other ctDNA-based screening methods under investigation include quantification of ctDNA, WGS, and targeted sequencing. Perego et al. found quantifying ctDNA with a 6.1 ng/mL plasma cutoff resulted in a 62.9% sensitive and 96.7% specific RCC detection test [147]. The ROC curve yielded a 0.761 AUC from the analysis of plasma DNA concentration [147]. Using WGS, Mouliere et al. detected RCC with 65% sensitivity and 95% specificity from plasma samples of 200 patients with different cancer diagnoses [148]. Bacon et al. utilized targeted sequencing of 981 cancer genes, reporting *VHL*, *BAP1*, and *PBRM1* to be the most common alterations in patients with metastatic disease, with a tissue tumor concordance of 77% [149]. Further validation efforts are crucial to determine these methods’ reliability and clinical applicability.

#### 3.3.2. Renal Cell Carcinoma Prognostics

Several studies have reported that quantification of ctDNA may predict OS and progression-free survival (PFS) in RCC. Yamamoto et al. found that ctDNA detection and short ctDNA fragment length were associated with shorter PFS and shorter cancer-specific survival for treatment-naïve patients with RCC [150]. For patients with metastasis, positive ctDNA, a short fragment size of ctDNA, and a high proportion of ctDNA predicted shorter cancer-specific survival [150]. Maia et al. assessed ctDNA in 34 RCC patients, reporting that detectable ctDNA was not associated with the International mRCC Database Consortium risk score or histology but did correlate to the sum of the longest diameters, used as a surrogate for tumor burden [151]. Bacon et al. report a shorter OS and PFS to first-line therapy for patients with detectable ctDNA [149].

DNA tumor methylation patterns may also predict tumor diameter, metastasis, and overall prognosis in RCC [152]. In ccRCC, *PCDH17* methylation was independently predictive of worse PFS (HR: 4.215) and OS (HR: 5.092) and significantly correlated to advanced stage disease (*p* = 0.044), high grades (*p* = 0.019), and lymph node metastasis (*p* = 0.008) [144]. De Martino et al. report an HR of 15.03 (*p* < 0.001) among patients with high *RNF185* methylation (>2400 GE/mL, 48.4% of patients) compared to low *RNF185* methylation [153]. However, the detection of *VHL*, *RASSF1A*, *P16*, and *PTGS2* methylation did not impact survival in a cohort of 200 pre-operative patients with RCC [153]. Jung et al. report *SHOX2* methylation in plasma samples correlated to advanced disease stage and risk of death post-nephrectomy [154].

Like in other GU cancers, ctDNA may reveal a more comprehensive and dynamic genomic profile of RCC. Hahn et al. found significant differences between the tumor-based FoundationOne assay and the ctDNA-based Guardant360 assay (*p* < 0.0001) [155]. The low concordance rates suggest that ctDNA may provide complementary information to standard-of-care tumor tissue genetic testing, potentially enhancing RCC prognostication.

#### 3.3.3. Renal Cell Carcinoma Therapeutic Implications

Due to the lower prevalence of patients with RCC and the typically lower quantifiable ctDNA compared to other GU cancers, studies exploring ctDNA to guide therapy stratification in RCC are more limited. In an analysis of sixty-nine patients with diverse malignancies, including three patients with RCC and two patients with bladder cancer, higher levels of variants of unknown significance and higher total alteration levels in ctDNA demonstrated significant improvements in PFS and OS in response to ICI [156]. Feng et al. quantified ctDNA in RCC longitudinally in patients receiving sorafenib. Higher levels of ctDNA were associated with disease progression; ctDNA measured at eight weeks predicted progression with 66.7% sensitivity and 100% specificity [157].

**Figure 2:**
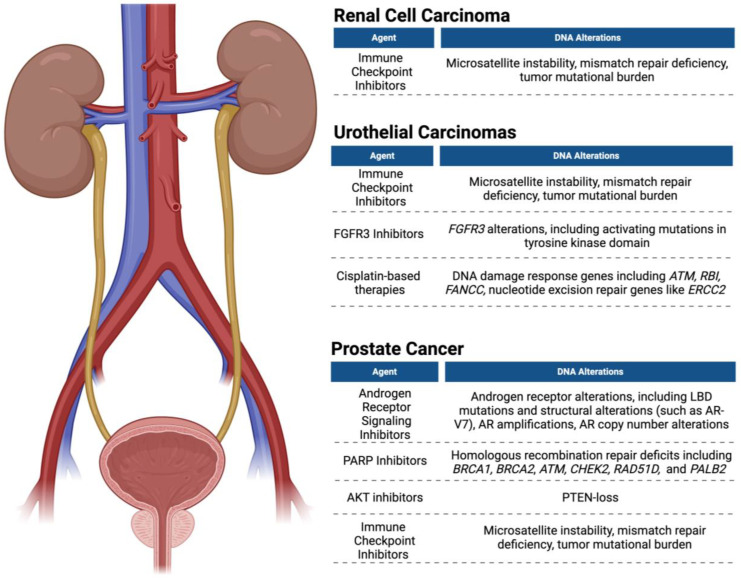
ctDNA detection and targeted therapies in renal cell carcinoma, urothelial carcinoma, and prostate cancer. Figure was created using BioRender.com (accessed on 18 June 2024).

Pal et al. studied ctDNA as a method of detecting genomic changes after RCC first-line regimens, including sunitinib, pazopanib, and bevacizumab, and post-first-line therapies, including everolimus, axitinib, cabozantinib, and nivolumab [158]. Genetic changes in ctDNA most frequently observed between first-line and subsequent agents included *TP53* (24% vs. 49%), *VHL* (18% vs. 29%), *NF1* (3% vs. 20%), *EGFR* (8% vs. 15%), and *PIK3CA* (8% vs. 17%) [158]. CtDNA alterations were most frequently observed after first-line treatment with VEGF inhibitors, which was remarkable for a 31% to 64% increase in *TP53* prevalence and a 4% to 29% increase in *NF1* detection [158]. These genetic alterations observed from ctDNA indicate possible mechanisms of disease resistance identified by ctDNA, which could inform therapy decisions after resistance to first-line agents [158]. Table 3 displays treatment options with the corresponding ctDNA-detectable target and the drug target in bladder cancer and RCC. Figure 2 displays ctDNA targets and respective targeted agents used for RCC, urothelial carcinomas, and prostate cancer.

#### 3.3.4. Renal Cell Carcinoma Resistance, Relapse, and Residual Disease Monitoring

In RCC, ctDNA has been utilized to detect disease recurrence post-nephrectomy. In a study of 30 patients with non-metastatic RCC undergoing nephrectomy, MSI was assessed using urine, serum, and tumor samples [159]. In a two-year follow-up, microsatellite alterations detected in preoperative serum were significantly associated with disease recurrence (*p* < 0.01), while no significant association was found between MSI detected in urine or tumor specimens [159]. Lasseter et al. found that serial ctDNA analysis showed VAF to be reduced in patients with a treatment response [160]. Wan et al. detected ctDNA via quantitative real-time PCR in patients with localized ccRCC, finding that patients with higher ctDNA had higher post-operative recurrence rates (*p* = 0.024) [161]. Pal et al. found significant differences in ctDNA from patients treated with conventional first-line agents compared to post-first-line agents, revealing potential mechanisms of treatment resistance. These studies represent distinct methods for using ctDNA to detect and predict disease resistance and relapse in RCC.

### 3.4. Penile, Adrenal, and Testicular Cancer

#### 3.4.1. Penile, Adrenal, and Testicular Cancer Detection

Screening methods for other GU cancers, including penile, adrenal, and testicular cancers, have received comparatively less research attention. Circulating HPV DNA has been investigated for its cancer detection potential in HPV-associated cancers. For instance, plasma HPV ctDNA has been shown to be a sensitive marker of HPV oropharyngeal, nasopharyngeal, and sinonasal squamous cell carcinoma, as well as in cervical cancers [162,163]. Although HPV has a role in an estimated 50.8% of penile cancers, the application of HPV ctDNA has yet to be studied in penile cancer [164,165].

Several approaches have been studied to detect testicular cancer via ctDNA. Ellinger et al. detected actin-β DNA fragments from 39 patients with seminomas, 35 patients with nonseminomatous testicular cancers, and 35 healthy individuals [166]. Actin-β detection distinguished testicular carcinoma patients from healthy individuals with an 87% sensitivity and 97% specificity [166]. CtDNA detection of testicular cancer remained high, with an 84% sensitivity and 97% specificity for patients without established testicular cancer biomarkers, including alpha-fetoprotein, human chorionic gonadotropin, placental alkaline phosphatase, and lactate dehydrogenase [166]. Detection of 79-bp mitochondrial DNA was a 59.5% sensitive and 94.3% specific test for detecting testicular cancer [167]. CtDNA hypermethylation at *APC*, *GSTP1*, *PTGS2*, *p14*, *p16*, and *RASSF1A* was investigated to detect seminomas and nonseminomas, with detected methylation patterns found to be similar between seminoma and nonseminomas [168]. When different methylation patterns were combined, ctDNA detected testicular cancer with 67% sensitivity and 97% specificity [168]. Raos et al. found methylation at CpG8/CpG9/CpG10 in *KITLG* to detect seminomas with 79% specificity and 71% sensitivity from blood samples [169]. These studies serve as proof of concept that ctDNA may be used to detect seminomas and nonseminomas; however, significant validation is needed.

#### 3.4.2. Penile, Adrenal, and Testicular Cancer Prognostics

To date, few studies have investigated ctDNA for use as a prognostic biomarker in other GU cancers. This challenge is underscored by the comparative rarity with which these cancers occur. However, this endeavor could play an important role in stratifying patients to treatment options based on personalized genetics. Nazha et al. successfully characterized genetic alterations in 80% of patients with adrenocortical carcinoma (ACC) using Guardant360 NGS [170]. CtDNA gene detection was concordant with mutations detected via tumor samples and analysis revealed clinically actionable alterations, including *EGFR*, *BRAF*, *MET*, *CDK4*, *CDKN2A*, *ATM*, and *CDK6* [170]. Although no genetic-based therapeutic options currently exist for ACC, trials and case reports have indicated the role of genetic biomarkers for novel applications of therapeutic options such as ICI [171,172]. In contrast, Garinet et al. reported detectable ctDNA in only 2 of 11 ACC patients, both of whom had metastatic disease [173]. Further investigation is required to explore the potential applications of ctDNA assays in rarer GU cancers.

## 4. Limitations and Future Directions

Although ctDNA has promise to improve the detection and management of GU cancers, there are significant limitations of ctDNA that hinder clinical implementation. High costs, low blood levels of ctDNA, limited sensitivity and specificity leading to false negative and positive tests, and other practical considerations challenge widespread clinical implementation. Threshold levels of ctDNA limit assay use for screening, particularly in early-stage prostate cancer and RCC, which have low fractions of plasma ctDNA. Low ctDNA shedding and low frequency mutations may falsely lead to negative results. Estimates from ctDNA in non-small-cell lung cancer and fetal/maternal DNA studies have been extrapolated to predict that with current ctDNA detection techniques, tumor burden would have to be between 1 and 3 cm in order to be detected by ctDNA assays, which is a size that would often be detectable by imaging [174]. CtDNA testing is further limited by the high false positive rates, which may create significant anxiety for patients and cause further harm through unnecessary invasive work-up. High false negative rates may conversely give patients a false reassurance. Clinical validity needs to be further demonstrated before implementing ctDNA in a clinical setting.

CtDNA analysis is further limited by the assumption that DNA will be transcribed to RNA and translated to a functional protein expressed by the cell. CtDNA may present too simplistic a representation of complex tumor gene expression. Identification of certain promoter elements and DNA fragmentation with ctDNA can predict gene expression [175,176]. Improving analysis methods that can reveal complex genomic profiles presents an important area of research.

Newer methylome-based analysis techniques may improve the utility of ctDNA screening by detecting early-cancer methylation changes at lower costs, but they need significant clinical validation [15]. Current technology in ctDNA epigenetic detection focuses on methylation, but other epigenetic modifications including phosphorylation, acetylation, and ubiquitination represent areas of further research and opportunities to improve sensitivity through combined epigenetic analysis in one single assay. Furthermore, precisely locating the site of the tumor or site metastasis can limit ctDNA utility. Methylation patterns have been demonstrated to help locate the origin of tumor detected by ctDNA [20]. Methods that can more precisely locate tumor and metastatic sites are an area for future research. The role of ctDNA in complement with proteomics and lipidomics as well as with imaging techniques represents an important area of research for clinical implementation of ctDNA.

CtDNA assay utility for GU cancers is currently of little clinical utility due to the high cost and absence of significant change in clinical management. In a multiplex PCR-based panel of ctDNA used in non-small-cell lung carcinoma, there is a current average cost of USD 1750 for single-region sequencing, the creation of a patient-specific panel, and sequencing of five samples [177]. NGS methods have an estimated 2–3 week turnaround time, which may limit the ability of ctDNA to predict tumor response before biopsy results or imaging, thus having a limited impact on clinical management [12]. CtDNA assays must show clinical benefits, such as improved patient outcomes, cost reduction, or greater ease of medical treatment for clinical implementation [178]. Although ctDNA holds great promise for the future of personalized GU cancer care, there is currently not sufficient evidence to justify ctDNA screening, prognosis, or therapeutic guidance in the management of GU cancers [178]. Investigation of the use of ctDNA in GU studies is rapidly expanding, which necessitates randomized clinical trials to determine the clinical utility of ctDNA in prognosis, treatment, and detection of relapse.

## 5. Conclusions

The use of ctDNA in GU cancers has multi-faceted applications and a promising future. Early detection and screening remain a significant unmet need in GU cancers, and meeting this need holds great potential to reduce cancer-related mortality and morbidity. As screening methods for GU cancers are widely debated or non-existent, ctDNA will likely change the landscape of disease detection biomarkers. As therapeutic options progress towards personalized medicine in which tumor molecular biology and genetics are imperative to decision-making, ctDNA can non-invasively detect genetic alterations and methylation patterns in real time. Several trials have investigated ctDNA as a genetic biomarker to predict subsets of patients who may have improved responses to genetically targeted therapies. Longitudinal ctDNA monitoring has facilitated the tracking of treatment-related genetic alterations, with compelling evidence supporting ctDNA’s ability to reveal AR alterations that contribute to therapy resistance in the context of ARSI treatment. In a post-resection setting, ctDNA may detect residual and recurrence disease before current clinical standards. In summary, CtDNA is a biomarker that may, with further development and validation, revolutionize the diagnosis, treatment, and post-treatment monitoring of GU cancers.

## Figures and Tables

**Table 1:** Technologies for CtDNA detection.

CtDNA Detection Method	Approach	Sensitivity	Potential Applications	Limitations
Droplet Digtal PCR (ddPCR)	Quantification of targeted genetic alterations [7,9]	High for pre-specified mutations [7,9]	Detecting targeted mutations, tracking treatment response, relapse, and recurrence	Omits genetic alterations or subclonal populations not pre-targeted
Beads, Emulsion, Amplification, and Magnetics (BEAMing)	Quantification of targeted genetic alterations [9]	High for pre-specified mutations [9]	Detecting targeted mutations, tracking treatment response, relapse, and recurrence	Omits genetic alterations or subclonal populations not targeted
Next-generation Sequencing (NGS)	Detection of a range of genetic alterations, including variant allele frequency, insertions, deletions, and structural variants [11]	High for a range of genetic alterations [11]	Identification of actionable or high-risk alterations, detection of alterations associated with mechanisms of treatment resistance, detection of tumor genetic evolution, detection of subclonal populations	High costs, multi-week turnaround time, data which may be difficult to interpret or ambiguous
Immunoprecipitation-based Epigenetic Targeted Assays	Detection of cancer-specific epigenetic patterns including DNA methylation, nucleosome positioning, and histone modification [14]	Variable [17,18]	Early cancer diagnosis and prognosis	Potentially lower sensitivity, challenges in interpreting data, greater technical challenges in interpretation of results

**Table 2:** CtDNA detection and targeted therapies in prostate cancer.

Agent	ctDNA Detectable Target	Drug Target	Reference
Androgen Receptor Signaling Inhibitors (Enzalutamide and Abiraterone)	Androgen receptor alterations, including LBD mutations and structural alterations (such as AR-V7), AR amplifications, and AR copy number alterations	Ligand binding domain of androgen receptor and downstream androgen receptor signaling	[53,58,59,60,61,62,63,64,65,66,67]
PARP Inhibitors (Olaparib, Rucaparib)	Homologous recombination repair deficits including *BRCA1*, *BRCA2*, *ATM*, *CHEK2*, *RAD51D*, and *PALB2*	Poly adenosine diphosphate (ADP)–ribose polymerase (PARP) inhibitor (single-strand DNA repair)	[69,70,71,72,73,74,75,76,77]
AKT inhibitors (Ipatasertib)	*PTEN* loss	PTEN-PI3K-AKT pathway	[78,79,80]
Immune Checkpoint Inhibitors (Pembrolizumab)	Microsatellite instability, mismatch repair deficiency, and tumor mutational burden	Anti-programmed cell death protein 1 (Anti-PD-1)	[81,82,84,85]

**Table 3:** CtDNA detection and targeted therapies in bladder cancer and renal cell carcinoma.

Agent	Genetic Target	Drug Target	Reference
Immune Modulatory Therapy (Pembrolizumab, Atezolizumab, Toripalimab, Ipilimumab, and Durvalumab)	Microsatellite instability, mismatch repair deficiency, tumor mutational burden	Anti-PD-1, Anti-PD-L1, Anti-CTLA-4	[118,119,120,121,122,124,156,157]
FGFR3 Inhibitors (Rogaratinib, Edafitinib, Furtibatinib, and Pemigatinib)	*FGFR3*	FGFR	[126,127,128]
Cisplatin-based Therapies	DNA damage response genes including *ATM*, *RBI*, and *FANCC*, nucleotide excision repair genes like *ERCC2*	Cross-links DNA	[129,130,131]

## Abbreviations

Circulating tumor DNA (ctDNA), genitourinary (GU), renal cell carcinoma (RCC), next-generation sequencing (NGS), droplet digital PCR (ddPCR), beads, emulsion, amplification, and magnetics (BEAMing), whole-genome sequencing (WGS), whole-exome sequencing (WES), prostate-specific antigen (PSA), receiver operating characteristic (ROC), area under the ROC curve (AUC), non-muscle-invasive bladder cancer (NMIBC), cell-free methylated DNA immunoprecipitation and high-throughput sequencing assay (cfMeDIP-seq), metastatic castration-resistant prostate cancer (mCRPC), androgen receptor (AR), microsatellite instability (MSI), overall survival (OS), hazard ratio (HR), time to progression (TTP), copy number (CN), variant allele frequency (VAF), failure-free survival (FFS), progression-free survival (PFS), adrenocortical carcinoma (ACC), immune checkpoint inhibitor (ICI), ligand binding domain (LBD), AR splice variant 7 (AR-V7), homologous recombination repair (HRR), overall response rate (ORR), DNA damage repair (DDR), homologous recombination deficiency (HRD), mismatch repair deficiency (dMMR), tumor mutational burden (TMB), poly adenosine diphosphate (ADP)–ribose polymerase (PARP), anti-programmed cell death protein 1 (Anti-PD-1), muscle-invasive bladder cancer (MIBC), disease-free survival (DFS), radical cystectomy (RC).

## References

[1] Wan J.C.M., Massie C., Garcia-Corbacho J., Mouliere F., Brenton J.D., Caldas C., Pacey S., Baird R., Rosenfeld N. (2017). Liquid biopsies come of age: Towards implementation of circulating tumour DNA. Nat. Rev. Cancer.

[2] Watanabe K., Nakamura Y., Low S.-K. (2021). Clinical implementation and current advancement of blood liquid biopsy in cancer. J. Hum. Genet..

[3] Bettegowda C., Sausen M., Leary R.J., Kinde I., Wang Y., Agrawal N., Bartlett B.R., Wang H., Luber B., Alani R.M. (2014). Detection of circulating tumor DNA in early- and late-stage human malignancies. Sci. Transl. Med..

[4] Rose K.M., Huelster H.L., Meeks J.J., Faltas B.M., Sonpavde G.P., Lerner S.P., Ross J.S., Spiess P.E., Grass G.D., Jain R.K. (2023). Circulating and urinary tumour DNA in urothelial carcinoma—Upper tract, lower tract and metastatic disease. Nat. Rev. Urol..

[5] Trujillo B., Wu A., Wetterskog D., Attard G. (2022). Blood-based liquid biopsies for prostate cancer: Clinical opportunities and challenges. Br. J. Cancer.

[6] Tivey A., Church M., Rothwell D., Dive C., Cook N. (2022). Circulating tumour DNA—Looking beyond the blood. Nat. Rev. Clin. Oncol..

[7] Odegaard J.I., Vincent J.J., Mortimer S., Vowles J.V., Ulrich B.C., Banks K.C., Fairclough S.R., Zill O.A., Sikora M., Mokhtari R. (2018). Validation of a Plasma-Based Comprehensive Cancer Genotyping Assay Utilizing Orthogonal Tissue- and Plasma-Based Methodologies. Clin. Cancer Res..

[8] García-Foncillas J., Alba E., Aranda E., Díaz-Rubio E., López-López R., Tabernero J., Vivancos A. (2017). Incorporating BEAMing technology as a liquid biopsy into clinical practice for the management of colorectal cancer patients: An expert taskforce review. Ann. Oncol..

[9] Nikanjam M., Kato S., Kurzrock R. (2022). Liquid biopsy: Current technology and clinical applications. J. Hematol. Oncol..

[10] Phallen J., Sausen M., Adleff V., Leal A., Hruban C., White J., Anagnostou V., Fiksel J., Cristiano S., Papp E. (2017). Direct detection of early-stage cancers using circulating tumor DNA. Sci. Transl. Med..

[11] Chen M., Zhao H. (2019). Next-generation sequencing in liquid biopsy: Cancer screening and early detection. Hum. Genom..

[12] Ewalt M.D., West H., Aisner D.L. (2019). Next Generation Sequencing—Testing Multiple Genetic Markers at Once. JAMA Oncol..

[13] García-Pardo M., Czarnecka-Kujawa K., Law J.H., Salvarrey A.M., Fernandes R., Fan Z.J., Waddell T.K., Yasufuku K., Liu G., Donahoe L.L. (2023). Association of Circulating Tumor DNA Testing before Tissue Diagnosis with Time to Treatment among Patients with Suspected Advanced Lung Cancer: The ACCELERATE Nonrandomized Clinical Trial. JAMA Netw. Open.

[14] Baca S.C., Seo J.-H., Davidsohn M.P., Fortunato B., Semaan K., Sotudian S., Lakshminarayanan G., Diossy M., Qiu X., El Zarif T. (2023). Liquid biopsy epigenomic profiling for cancer subtyping. Nat. Med..

[15] Shen S.Y., Singhania R., Fehringer G., Chakravarthy A., Roehrl M.H.A., Chadwick D., Zuzarte P.C., Borgida A., Wang T.T., Li T. (2018). Sensitive tumour detection and classification using plasma cell-free DNA methylomes. Nature.

[16] Liu M.C., Oxnard G.R., Klein E.A., Swanton C., Seiden M.V. (2020). Sensitive and specific multi-cancer detection and localization using methylation signatures in cell-free DNA. Ann. Oncol..

[17] Flores B.C.T., Correia M.P., Rodríguez J.G., Henrique R., Jerónimo C. (2021). Bridging the Gaps between Circulating Tumor Cells and DNA Methylation in Prostate Cancer. Cancers.

[18] Nassar F.J., Msheik Z.S., Nasr R.R., Temraz S.N. (2021). Methylated circulating tumor DNA as a biomarker for colorectal cancer diagnosis, prognosis, and prediction. Clin. Epigenet..

[19] Guo S., Diep D., Plongthongkum N., Fung H.L., Zhang K., Zhang K. (2017). Identification of methylation haplotype blocks aids in deconvolution of heterogeneous tissue samples and tumor tissue-of-origin mapping from plasma DNA. Nat. Genet..

[20] Hao X., Luo H., Krawczyk M., Wei W., Wang W., Wang J., Flagg K., Hou J., Zhang H., Yi S. (2017). DNA methylation markers for diagnosis and prognosis of common cancers. Proc. Natl. Acad. Sci. USA.

[21] Lorente D., Omlin A., Zafeiriou Z., Nava-Rodrigues D., Pérez-López R., Pezaro C., Mehra N., Sheridan E., Figueiredo I., Riisnaes R. (2016). Castration-Resistant Prostate Cancer Tissue Acquisition From Bone Metastases for Molecular Analyses. Clin. Genitourin. Cancer.

[22] Gerlinger M., Rowan A.J., Horswell S., Larkin J., Endesfelder D., Gronroos E., Martinez P., Matthews N., Stewart A., Tarpey P. (2012). Intratumor Heterogeneity and Branched Evolution Revealed by Multiregion Sequencing. N. Engl. J. Med..

[23] Bardelli A., Pantel K. (2017). Liquid Biopsies, What We Do Not Know (Yet). Cancer Cell.

[24] Clinton T.N., Chen Z., Wise H., Lenis A.T., Chavan S., Donoghue M.T.A., Almassi N., Chu C.E., Dason S., Rao P. (2022). Genomic heterogeneity as a barrier to precision oncology in urothelial cancer. Cell Rep..

[25] Herberts C., Annala M., Sipola J., Ng S.W.S., Chen X.E., Nurminen A., Korhonen O.V., Munzur A.D., Beja K., Schönlau E. (2022). Deep whole-genome ctDNA chronology of treatment-resistant prostate cancer. Nature.

[26] Wyatt A.W., Annala M., Aggarwal R., Beja K., Feng F., Youngren J., Foye A., Lloyd P., Nykter M., Beer T.M. (2017). Concordance of Circulating Tumor DNA and Matched Metastatic Tissue Biopsy in Prostate Cancer. J. Natl. Cancer Inst..

[27] Wolf A.M., Wender R.C., Etzioni R.B., Thompson I.M., D’Amico A.V., Volk R.J., Brooks D.D., Dash C., Guessous I., Andrews K. (2010). American Cancer Society guideline for the early detection of prostate cancer: Update 2010. CA Cancer J Clin.

[28] Kilpeläinen T.P., Tammela T.L., Roobol M., Hugosson J., Ciatto S., Nelen V., Moss S., Määttänen L., Auvinen A. (2011). False-positive screening results in the European randomized study of screening for prostate cancer. Eur. J. Cancer.

[29] Loeb S., Vellekoop A., Ahmed H.U., Catto J., Emberton M., Nam R., Rosario D.J., Scattoni V., Lotan Y. (2013). Systematic review of complications of prostate biopsy. Eur. Urol..

[30] Ahmed H.U., El-Shater Bosaily A., Brown L.C., Gabe R., Kaplan R., Parmar M.K., Collaco-Moraes Y., Ward K., Hindley R.G., Freeman A. (2017). Diagnostic accuracy of multi-parametric MRI and TRUS biopsy in prostate cancer (PROMIS): A paired validating confirmatory study. Lancet.

[31] Aravanis A.M., Lee M., Klausner R.D. (2017). Next-Generation Sequencing of Circulating Tumor DNA for Early Cancer Detection. Cell.

[32] Hennigan S.T., Trostel S.Y., Terrigino N.T., Voznesensky O.S., Schaefer R.J., Whitlock N.C., Wilkinson S., Carrabba N.V., Atway R., Shema S. (2019). Low Abundance of Circulating Tumor DNA in Localized Prostate Cancer. JCO Precis. Oncol..

[33] Berman B.P., Weisenberger D.J., Aman J.F., Hinoue T., Ramjan Z., Liu Y., Noushmehr H., Lange C.P.E., van Dijk C.M., Tollenaar R.A.E.M. (2012). Regions of focal DNA hypermethylation and long-range hypomethylation in colorectal cancer coincide with nuclear lamina–associated domains. Nat. Genet..

[34] Guo H., Vuille J.A., Wittner B.S., Lachtara E.M., Hou Y., Lin M., Zhao T., Raman A.T., Russell H.C., Reeves B.A. (2023). DNA hypomethylation silences anti-tumor immune genes in early prostate cancer and CTCs. Cell.

[35] Klein E.A., Richards D., Cohn A., Tummala M., Lapham R., Cosgrove D., Chung G., Clement J., Gao J., Hunkapiller N. (2021). Clinical validation of a targeted methylation-based multi-cancer early detection test using an independent validation set. Ann. Oncol..

[36] Hubbell E., Clarke C.A., Aravanis A.M., Berg C.D. (2021). Modeled Reductions in Late-stage Cancer with a Multi-Cancer Early Detection Test. Cancer Epidemiol. Biomark. Prev..

[37] Ellinger J., Haan K., Heukamp L.C., Kahl P., Büttner R., Müller S.C., von Ruecker A., Bastian P.J. (2008). CpG island hypermethylation in cell-free serum DNA identifies patients with localized prostate cancer. Prostate.

[38] Brikun I., Nusskern D., Decatus A., Harvey E., Li L., Freije D. (2018). A panel of DNA methylation markers for the detection of prostate cancer from FV and DRE urine DNA. Clin. Epigenet..

[39] Haldrup C., Pedersen A.L., Øgaard N., Strand S.H., Høyer S., Borre M., Ørntoft T.F., Sørensen K.D. (2018). Biomarker potential of ST6GALNAC3 and ZNF660 promoter hypermethylation in prostate cancer tissue and liquid biopsies. Mol. Oncol..

[40] Constâncio V., Nunes S.P., Moreira-Barbosa C., Freitas R., Oliveira J., Pousa I., Oliveira J., Soares M., Dias C.G., Dias T. (2019). Early detection of the major male cancer types in blood-based liquid biopsies using a DNA methylation panel. Clin. Epigenet..

[41] Reis I.M., Ramachandran K., Speer C., Gordian E., Singal R. (2015). Serum GADD45a methylation is a useful biomarker to distinguish benign vs malignant prostate disease. Br. J. Cancer.

[42] O’Reilly E., Tuzova A.V., Walsh A.L., Russell N.M., O’Brien O., Kelly S., Dhomhnallain O.N., DeBarra L., Dale C.M., Brugman R. (2019). epiCaPture: A Urine DNA Methylation Test for Early Detection of Aggressive Prostate Cancer. JCO Precis. Oncol..

[43] Henrique R., Jerónimo C. (2004). Molecular detection of prostate cancer: A role for GSTP1 hypermethylation. Eur. Urol..

[44] Wu T., Giovannucci E., Welge J., Mallick P., Tang W.Y., Ho S.M. (2011). Measurement of GSTP1 promoter methylation in body fluids may complement PSA screening: A meta-analysis. Br. J. Cancer.

[45] Helzer K.T., Sharifi M.N., Sperger J.M., Shi Y., Annala M., Bootsma M.L., Reese S.R., Taylor A., Kaufmann K.R., Krause H.K. (2023). Fragmentomic analysis of circulating tumor DNA-targeted cancer panels. Ann. Oncol..

[46] Spratt D.E., Yousefi K., Deheshi S., Ross A.E., Den R.B., Schaeffer E.M., Trock B.J., Zhang J., Glass A.G., Dicker A.P. (2017). Individual Patient-Level Meta-Analysis of the Performance of the Decipher Genomic Classifier in High-Risk Men after Prostatectomy to Predict Development of Metastatic Disease. J. Clin. Oncol..

[47] Spratt D.E., Huang H.-C., Michalski J.M., Davicioni E., Berlin A., Simko J., Efstathiou J.A., Tran P.T., Thompson D., Parliament M. (2022). Validation of the performance of the Decipher biopsy genomic classifier in intermediate-risk prostate cancer on the phase III randomized trial NRG Oncology/RTOG 0126. J. Clin. Oncol..

[48] Robinson D., Van Allen E.M., Wu Y.M., Schultz N., Lonigro R.J., Mosquera J.M., Montgomery B., Taplin M.E., Pritchard C.C., Attard G. (2015). Integrative clinical genomics of advanced prostate cancer. Cell.

[49] van Dessel L.F., van Riet J., Smits M., Zhu Y., Hamberg P., van der Heijden M.S., Bergman A.M., van Oort I.M., de Wit R., Voest E.E. (2019). The genomic landscape of metastatic castration-resistant prostate cancers reveals multiple distinct genotypes with potential clinical impact. Nat. Commun..

[50] Sonpavde G., Agarwal N., Pond G.R., Nagy R.J., Nussenzveig R.H., Hahn A.W., Sartor O., Gourdin T.S., Nandagopal L., Ledet E.M. (2019). Circulating tumor DNA alterations in patients with metastatic castration-resistant prostate cancer. Cancer.

[51] Tukachinsky H., Madison R.W., Chung J.H., Gjoerup O.V., Severson E.A., Dennis L., Fendler B.J., Morley S., Zhong L., Graf R.P. (2021). Genomic Analysis of Circulating Tumor DNA in 3,334 Patients with Advanced Prostate Cancer Identifies Targetable BRCA Alterations and AR Resistance Mechanisms. Clin. Cancer Res..

[52] Annala M., Vandekerkhove G., Khalaf D., Taavitsainen S., Beja K., Warner E.W., Sunderland K., Kollmannsberger C., Eigl B.J., Finch D. (2018). Circulating Tumor DNA Genomics Correlate with Resistance to Abiraterone and Enzalutamide in Prostate Cancer. Cancer Discov..

[53] Annala M., Taavitsainen S., Khalaf D.J., Vandekerkhove G., Beja K., Sipola J., Warner E.W., Herberts C., Wong A., Fu S. (2021). Evolution of Castration-Resistant Prostate Cancer in ctDNA during Sequential Androgen Receptor Pathway Inhibition. Clin. Cancer Res..

[54] Romanel A., Gasi Tandefelt D., Conteduca V., Jayaram A., Casiraghi N., Wetterskog D., Salvi S., Amadori D., Zafeiriou Z., Rescigno P. (2015). Plasma AR and abiraterone-resistant prostate cancer. Sci. Transl. Med..

[55] Chi K.N., Annala M., Sunderland K., Khalaf D., Finch D., Oja C.D., Vergidis J., Zulfiqar M., Beja K., Vandekerkhove G. (2017). A randomized phase II cross-over study of abiraterone + prednisone (ABI) vs enzalutamide (ENZ) for patients (pts) with metastatic, castration-resistant prostate cancer (mCRPC). J. Clin. Oncol..

[56] Rebello R.J., Oing C., Knudsen K.E., Loeb S., Johnson D.C., Reiter R.E., Gillessen S., Van der Kwast T., Bristow R.G. (2021). Prostate cancer. Nat. Rev. Dis. Primers.

[57] Sato Y. (2022). Clinical utility of liquid biopsy-based companion diagnostics in the non-small-cell lung cancer treatment. Explor. Target. Antitumor Ther..

[58] Antonarakis E.S., Lu C., Wang H., Luber B., Nakazawa M., Roeser J.C., Chen Y., Mohammad T.A., Chen Y., Fedor H.L. (2014). AR-V7 and resistance to enzalutamide and abiraterone in prostate cancer. N. Engl. J. Med..

[59] Ryan C.J., Smith M.R., de Bono J.S., Molina A., Logothetis C.J., de Souza P., Fizazi K., Mainwaring P., Piulats J.M., Ng S. (2013). Abiraterone in metastatic prostate cancer without previous chemotherapy. N. Engl. J. Med..

[60] Scher H.I., Fizazi K., Saad F., Taplin M.E., Sternberg C.N., Miller K., de Wit R., Mulders P., Chi K.N., Shore N.D. (2012). Increased survival with enzalutamide in prostate cancer after chemotherapy. N. Engl. J. Med..

[61] O’Donnell A., Judson I., Dowsett M., Raynaud F., Dearnaley D., Mason M., Harland S., Robbins A., Halbert G., Nutley B. (2004). Hormonal impact of the 17alpha-hydroxylase/C(17,20)-lyase inhibitor abiraterone acetate (CB7630) in patients with prostate cancer. Br. J. Cancer.

[62] Scher H.I., Beer T.M., Higano C.S., Anand A., Taplin M.E., Efstathiou E., Rathkopf D., Shelkey J., Yu E.Y., Alumkal J. (2010). Antitumour activity of MDV3100 in castration-resistant prostate cancer: A phase 1-2 study. Lancet.

[63] Quigley D.A., Dang H.X., Zhao S.G., Lloyd P., Aggarwal R., Alumkal J.J., Foye A., Kothari V., Perry M.D., Bailey A.M. (2018). Genomic Hallmarks and Structural Variation in Metastatic Prostate Cancer. Cell.

[64] Lorente D., Mateo J., Zafeiriou Z., Smith A.D., Sandhu S., Ferraldeschi R., de Bono J.S. (2015). Switching and withdrawing hormonal agents for castration-resistant prostate cancer. Nat. Rev. Urol..

[65] Armstrong A.J., Halabi S., Luo J., Nanus D.M., Giannakakou P., Szmulewitz R.Z., Danila D.C., Healy P., Anand M., Rothwell C.J. (2019). Prospective Multicenter Validation of Androgen Receptor Splice Variant 7 and Hormone Therapy Resistance in High-Risk Castration-Resistant Prostate Cancer: The PROPHECY Study. J. Clin. Oncol..

[66] Conteduca V., Wetterskog D., Sharabiani M.T.A., Grande E., Fernandez-Perez M.P., Jayaram A., Salvi S., Castellano D., Romanel A., Lolli C. (2017). Androgen receptor gene status in plasma DNA associates with worse outcome on enzalutamide or abiraterone for castration-resistant prostate cancer: A multi-institution correlative biomarker study. Ann. Oncol..

[67] De Laere B., van Dam P.J., Whitington T., Mayrhofer M., Diaz E.H., Van den Eynden G., Vandebroek J., Del-Favero J., Van Laere S., Dirix L. (2017). Comprehensive Profiling of the Androgen Receptor in Liquid Biopsies from Castration-resistant Prostate Cancer Reveals Novel Intra-AR Structural Variation and Splice Variant Expression Patterns. Eur. Urol..

[68] Swami U., Zimmerman R.M., Nussenzveig R.H., Hernandez E.J., Jo Y., Sayegh N., Wesolowski S., Kiedrowski L.A., Barata P.C., Lemmon G.H. (2022). Genomic landscape of advanced prostate cancer patients with BRCA1 versus BRCA2 mutations as detected by comprehensive genomic profiling of cell-free DNA. Front. Oncol..

[69] Pritchard C.C., Mateo J., Walsh M.F., De Sarkar N., Abida W., Beltran H., Garofalo A., Gulati R., Carreira S., Eeles R. (2016). Inherited DNA-Repair Gene Mutations in Men with Metastatic Prostate Cancer. N. Engl. J. Med..

[70] Abida W., Patnaik A., Campbell D., Shapiro J., Bryce A.H., McDermott R., Sautois B., Vogelzang N.J., Bambury R.M., Voog E. (2020). Rucaparib in Men with Metastatic Castration-Resistant Prostate Cancer Harboring a BRCA1 or BRCA2 Gene Alteration. J. Clin. Oncol..

[71] Hussain M., Mateo J., Fizazi K., Saad F., Shore N., Sandhu S., Chi K.N., Sartor O., Agarwal N., Olmos D. (2020). Survival with Olaparib in Metastatic Castration-Resistant Prostate Cancer. N. Engl. J. Med..

[72] Mateo J., Carreira S., Sandhu S., Miranda S., Mossop H., Perez-Lopez R., Nava Rodrigues D., Robinson D., Omlin A., Tunariu N. (2015). DNA-Repair Defects and Olaparib in Metastatic Prostate Cancer. N. Engl. J. Med..

[73] Chi K.N., Barnicle A., Sibilla C., Lai Z., Corcoran C., Barrett J.C., Adelman C.A., Qiu P., Easter A., Dearden S. (2023). Detection of BRCA1, BRCA2, and ATM Alterations in Matched Tumor Tissue and Circulating Tumor DNA in Patients with Prostate Cancer Screened in PROfound. Clin. Cancer Res..

[74] Goodall J., Mateo J., Yuan W., Mossop H., Porta N., Miranda S., Perez-Lopez R., Dolling D., Robinson D.R., Sandhu S. (2017). Circulating Cell-Free DNA to Guide Prostate Cancer Treatment with PARP Inhibition. Cancer Discov..

[75] Quigley D., Alumkal J.J., Wyatt A.W., Kothari V., Foye A., Lloyd P., Aggarwal R., Kim W., Lu E., Schwartzman J. (2017). Analysis of Circulating Cell-Free DNA Identifies Multiclonal Heterogeneity of BRCA2 Reversion Mutations Associated with Resistance to PARP Inhibitors. Cancer Discov..

[76] Anscher M.S., Chang E., Gao X., Gong Y., Weinstock C., Bloomquist E., Adeniyi O., Charlab R., Zimmerman S., Serlemitsos-Day M. (2021). FDA Approval Summary: Rucaparib for the Treatment of Patients with Deleterious BRCA-Mutated Metastatic Castrate-Resistant Prostate Cancer. Oncologist.

[77] Woodhouse R., Li M., Hughes J., Delfosse D., Skoletsky J., Ma P., Meng W., Dewal N., Milbury C., Clark T. (2020). Clinical and analytical validation of FoundationOne Liquid CDx, a novel 324-Gene cfDNA-based comprehensive genomic profiling assay for cancers of solid tumor origin. PLoS ONE.

[78] Sweeney C., Bracarda S., Sternberg C.N., Chi K.N., Olmos D., Sandhu S., Massard C., Matsubara N., Alekseev B., Parnis F. (2021). Ipatasertib plus abiraterone and prednisolone in metastatic castration-resistant prostate cancer (IPATential150): A multicentre, randomised, double-blind, phase 3 trial. Lancet.

[79] Bang S., Won D., Shin S., Cho K.S., Park J.W., Lee J., Choi Y.D., Kang S., Lee S.T., Choi J.R. (2023). Circulating Tumor DNA Analysis on Metastatic Prostate Cancer with Disease Progression. Cancers.

[80] Dong X., Zheng T., Zhang M., Dai C., Wang L., Wang L., Zhang R., Long Y., Wen D., Xie F. (2021). Circulating Cell-Free DNA-Based Detection of Tumor Suppressor Gene Copy Number Loss and Its Clinical Implication in Metastatic Prostate Cancer. Front. Oncol..

[81] Lanka S.M., Zorko N.A., Antonarakis E.S., Barata P.C. (2023). Metastatic Castration-Resistant Prostate Cancer, Immune Checkpoint Inhibitors, and Beyond. Curr. Oncol..

[82] Marabelle A., Le D.T., Ascierto P.A., Giacomo A.M.D., Jesus-Acosta A.D., Delord J.-P., Geva R., Gottfried M., Penel N., Hansen A.R. (2020). Efficacy of Pembrolizumab in Patients with Noncolorectal High Microsatellite Instability/Mismatch Repair–Deficient Cancer: Results From the Phase II KEYNOTE-158 Study. J. Clin. Oncol..

[83] Muzny D.M., Bainbridge M.N., Chang K., Dinh H.H., Drummond J.A., Fowler G., Kovar C.L., Lewis L.R., Morgan M.B., Newsham I.F. (2012). Comprehensive molecular characterization of human colon and rectal cancer. Nature.

[84] Dudley J.C., Lin M.T., Le D.T., Eshleman J.R. (2016). Microsatellite Instability as a Biomarker for PD-1 Blockade. Clin. Cancer Res..

[85] Abida W., Cheng M.L., Armenia J., Middha S., Autio K.A., Vargas H.A., Rathkopf D., Morris M.J., Danila D.C., Slovin S.F. (2019). Analysis of the Prevalence of Microsatellite Instability in Prostate Cancer and Response to Immune Checkpoint Blockade. JAMA Oncol..

[86] Black J.R.M., McGranahan N. (2021). Genetic and non-genetic clonal diversity in cancer evolution. Nat. Rev. Cancer.

[87] Wyatt A.W., Azad A.A., Volik S.V., Annala M., Beja K., McConeghy B., Haegert A., Warner E.W., Mo F., Brahmbhatt S. (2016). Genomic Alterations in Cell-Free DNA and Enzalutamide Resistance in Castration-Resistant Prostate Cancer. JAMA Oncol..

[88] Lallous N., Volik S.V., Awrey S., Leblanc E., Tse R., Murillo J., Singh K., Azad A.A., Wyatt A.W., LeBihan S. (2016). Functional analysis of androgen receptor mutations that confer anti-androgen resistance identified in circulating cell-free DNA from prostate cancer patients. Genome Biol..

[89] Tolmeijer S.H., Boerrigter E., Sumiyoshi T., Kwan E.M., Ng S.W.S., Annala M., Donnellan G., Herberts C., Benoist G.E., Hamberg P. (2023). Early On-treatment Changes in Circulating Tumor DNA Fraction and Response to Enzalutamide or Abiraterone in Metastatic Castration-Resistant Prostate Cancer. Clin. Cancer Res..

[90] Conteduca V., Wetterskog D., Scarpi E., Romanel A., Gurioli G., Jayaram A., Lolli C., Tandefelt D.G., Schepisi G., Casadei C. (2020). Plasma tumour DNA as an early indicator of treatment response in metastatic castration-resistant prostate cancer. Br. J. Cancer.

[91] Jayaram A., Wingate A., Wetterskog D., Wheeler G., Sternberg C.N., Jones R., Berruti A., Lefresne F., Lahaye M., Thomas S. (2021). Plasma tumor gene conversions after one cycle abiraterone acetate for metastatic castration-resistant prostate cancer: A biomarker analysis of a multicenter international trial. Ann. Oncol..

[92] Silva R., Moran B., Baird A.M., O’Rourke C.J., Finn S.P., McDermott R., Watson W., Gallagher W.M., Brennan D.J., Perry A.S. (2021). Longitudinal analysis of individual cfDNA methylome patterns in metastatic prostate cancer. Clin. Epigenet..

[93] Tessneer K.L., Pasula S., Cai X., Dong Y., Liu X., Yu L., Hahn S., McManus J., Chen Y., Chang B. (2013). Endocytic adaptor protein epsin is elevated in prostate cancer and required for cancer progression. ISRN Oncol..

[94] Mahon K.L., Qu W., Lin H.M., Spielman C., Cain D., Jacobs C., Stockler M.R., Higano C.S., de Bono J.S., Chi K.N. (2019). Serum Free Methylated Glutathione S-transferase 1 DNA Levels, Survival, and Response to Docetaxel in Metastatic, Castration-resistant Prostate Cancer: Post Hoc Analyses of Data from a Phase 3 Trial. Eur. Urol..

[95] Birkenkamp-Demtröder K., Nordentoft I., Christensen E., Høyer S., Reinert T., Vang S., Borre M., Agerbæk M., Jensen J.B., Ørntoft T.F. (2016). Genomic Alterations in Liquid Biopsies from Patients with Bladder Cancer. Eur. Urol..

[96] Agarwal N., Pal S.K., Hahn A.W., Nussenzveig R.H., Pond G.R., Gupta S.V., Wang J., Bilen M.A., Naik G., Ghatalia P. (2018). Characterization of metastatic urothelial carcinoma via comprehensive genomic profiling of circulating tumor DNA. Cancer.

[97] Jain M., Kamalov D., Tivtikyan A., Balatsky A., Samokhodskaya L., Okhobotov D., Kozlova P., Pisarev E., Zvereva M., Kamalov A. (2021). Urine TERT promoter mutations-based tumor DNA detection in patients with bladder cancer: A pilot study. Mol. Clin. Oncol..

[98] Dudley J.C., Schroers-Martin J., Lazzareschi D.V., Shi W.Y., Chen S.B., Esfahani M.S., Trivedi D., Chabon J.J., Chaudhuri A.A., Stehr H. (2019). Detection and Surveillance of Bladder Cancer Using Urine Tumor DNA. Cancer Discov..

[99] Xu Y., Kim Y.H., Jeong P., Piao X.M., Byun Y.J., Kang H.W., Kim W.T., Lee J.Y., Kim I.Y., Moon S.K. (2019). Diagnostic value of combined IQGAP3/BMP4 and IQGAP3/FAM107A expression ratios in urinary cell-free DNA for discriminating bladder cancer from hematuria. Urol. Oncol..

[100] Cheng T.H.T., Jiang P., Teoh J.Y.C., Heung M.M.S., Tam J.C.W., Sun X., Lee W.S., Ni M., Chan R.C.K., Ng C.F. (2019). Noninvasive Detection of Bladder Cancer by Shallow-Depth Genome-Wide Bisulfite Sequencing of Urinary Cell-Free DNA for Methylation and Copy Number Profiling. Clin. Chem..

[101] Xu Y., Ma X., Ai X., Gao J., Liang Y., Zhang Q., Ma T., Mao K., Zheng Q., Wang S. (2020). A Urine-Based Liquid Biopsy Method for Detection of Upper Tract Urinary Carcinoma. Front. Oncol..

[102] Zeng S., Ying Y., Xing N., Wang B., Qian Z., Zhou Z., Zhang Z., Xu W., Wang H., Dai L. (2020). Noninvasive Detection of Urothelial Carcinoma by Cost-effective Low-coverage Whole-genome Sequencing from Urine-Exfoliated Cell DNA. Clin. Cancer Res..

[103] Christensen E., Birkenkamp-Demtröder K., Sethi H., Shchegrova S., Salari R., Nordentoft I., Wu H.-T., Knudsen M., Lamy P., Lindskrog S.V. (2019). Early Detection of Metastatic Relapse and Monitoring of Therapeutic Efficacy by Ultra-Deep Sequencing of Plasma Cell-Free DNA in Patients with Urothelial Bladder Carcinoma. J. Clin. Oncol..

[104] Christensen E., Birkenkamp-Demtröder K., Nordentoft I., Høyer S., van der Keur K., van Kessel K., Zwarthoff E., Agerbæk M., Ørntoft T.F., Jensen J.B. (2017). Liquid Biopsy Analysis of FGFR3 and PIK3CA Hotspot Mutations for Disease Surveillance in Bladder Cancer. Eur. Urol..

[105] Shohdy K.S., Villamar D.M., Cao Y., Trieu J., Price K.S., Nagy R., Tagawa S.T., Molina A.M., Sternberg C.N., Nanus D.M. (2022). Serial ctDNA analysis predicts clinical progression in patients with advanced urothelial carcinoma. Br. J. Cancer.

[106] Grivas P., Lalani A.A., Pond G.R., Nagy R.J., Faltas B., Agarwal N., Gupta S.V., Drakaki A., Vaishampayan U.N., Wang J. (2020). Circulating Tumor DNA Alterations in Advanced Urothelial Carcinoma and Association with Clinical Outcomes: A Pilot Study. Eur. Urol. Oncol..

[107] Vandekerkhove G., Todenhöfer T., Annala M., Struss W.J., Wong A., Beja K., Ritch E., Brahmbhatt S., Volik S.V., Hennenlotter J. (2017). Circulating Tumor DNA Reveals Clinically Actionable Somatic Genome of Metastatic Bladder Cancer. Clin. Cancer Res..

[108] Puntoni M., Petrera M., Campora S., Garrone E., Defferrari C., Torrisi R., Johansson H., Bruno S., Curotto A., DeCensi A. (2016). Prognostic Significance of VEGF after Twenty-Year Follow-up in a Randomized Trial of Fenretinide in Non–Muscle-Invasive Bladder Cancer. Cancer Prev. Res..

[109] Christensen E., Nordentoft I., Birkenkamp-Demtröder K., Elbæk S.K., Lindskrog S.V., Taber A., Andreasen T.G., Strandgaard T., Knudsen M., Lamy P. (2023). Cell-Free Urine and Plasma DNA Mutational Analysis Predicts Neoadjuvant Chemotherapy Response and Outcome in Patients with Muscle-Invasive Bladder Cancer. Clin. Cancer Res..

[110] Powles T., Assaf Z.J., Davarpanah N., Banchereau R., Szabados B.E., Yuen K.C., Grivas P., Hussain M., Oudard S., Gschwend J.E. (2021). ctDNA guiding adjuvant immunotherapy in urothelial carcinoma. Nature.

[111] Robertson A.G., Kim J., Al-Ahmadie H., Bellmunt J., Guo G., Cherniack A.D., Hinoue T., Laird P.W., Hoadley K.A., Akbani R. (2017). Comprehensive Molecular Characterization of Muscle-Invasive Bladder Cancer. Cell.

[112] Ross J.S., Wang K., Khaira D., Ali S.M., Fisher H.A., Mian B., Nazeer T., Elvin J.A., Palma N., Yelensky R. (2016). Comprehensive genomic profiling of 295 cases of clinically advanced urothelial carcinoma of the urinary bladder reveals a high frequency of clinically relevant genomic alterations. Cancer.

[113] Vandekerkhove G., Lavoie J.-M., Annala M., Murtha A.J., Sundahl N., Walz S., Sano T., Taavitsainen S., Ritch E., Fazli L. (2021). Plasma ctDNA is a tumor tissue surrogate and enables clinical-genomic stratification of metastatic bladder cancer. Nat. Commun..

[114] Barata P.C., Koshkin V.S., Funchain P., Sohal D., Pritchard A., Klek S., Adamowicz T., Gopalakrishnan D., Garcia J., Rini B. (2017). Next-generation sequencing (NGS) of cell-free circulating tumor DNA and tumor tissue in patients with advanced urothelial cancer: A pilot assessment of concordance. Ann. Oncol..

[115] Klempner S.J., Fabrizio D., Bane S., Reinhart M., Peoples T., Ali S.M., Sokol E.S., Frampton G., Schrock A.B., Anhorn R. (2020). Tumor Mutational Burden as a Predictive Biomarker for Response to Immune Checkpoint Inhibitors: A Review of Current Evidence. Oncologist.

[116] Alexandrov L.B., Nik-Zainal S., Wedge D.C., Aparicio S.A.J.R., Behjati S., Biankin A.V., Bignell G.R., Bolli N., Borg A., Børresen-Dale A.-L. (2013). Signatures of mutational processes in human cancer. Nature.

[117] Maia M.C., Salgia M., Pal S.K. (2020). Harnessing cell-free DNA: Plasma circulating tumour DNA for liquid biopsy in genitourinary cancers. Nat. Rev. Urol..

[118] Rosenberg J.E., Hoffman-Censits J., Powles T., van der Heijden M.S., Balar A.V., Necchi A., Dawson N., O’Donnell P.H., Balmanoukian A., Loriot Y. (2016). Atezolizumab in patients with locally advanced and metastatic urothelial carcinoma who have progressed following treatment with platinum-based chemotherapy: A single-arm, multicentre, phase 2 trial. Lancet.

[119] Necchi A., Anichini A., Raggi D., Briganti A., Massa S., Lucianò R., Colecchia M., Giannatempo P., Mortarini R., Bianchi M. (2018). Pembrolizumab as Neoadjuvant Therapy before Radical Cystectomy in Patients with Muscle-Invasive Urothelial Bladder Carcinoma (PURE-01): An Open-Label, Single-Arm, Phase II Study. J. Clin. Oncol..

[120] Sheng X., Chen H., Hu B., Yao X., Liu Z., Yao X., Guo H., Hu Y., Ji Z., Luo H. (2022). Safety, Efficacy, and Biomarker Analysis of Toripalimab in Patients with Previously Treated Advanced Urothelial Carcinoma: Results from a Multicenter Phase II Trial POLARIS-03. Clin. Cancer Res..

[121] Zang J., Zhang R., Jin D., Xie F., Shahatiaili A., Wu G., Zhang Y., Zhao Z., Du P., Jia S. (2023). Integrated longitudinal circulating tumor DNA profiling predicts immunotherapy response of metastatic urothelial carcinoma in the POLARIS-03 trial. J. Pathol..

[122] Szabados B., Kockx M., Assaf Z.J., van Dam P.J., Rodriguez-Vida A., Duran I., Crabb S.J., Van Der Heijden M.S., Pous A.F., Gravis G. (2022). Final Results of Neoadjuvant Atezolizumab in Cisplatin-ineligible Patients with Muscle-invasive Urothelial Cancer of the Bladder. Eur. Urol..

[123] Raja R., Kuziora M., Brohawn P.Z., Higgs B.W., Gupta A., Dennis P.A., Ranade K. (2018). Early Reduction in ctDNA Predicts Survival in Patients with Lung and Bladder Cancer Treated with Durvalumab. Clin. Cancer Res..

[124] van Dorp J., Pipinikas C., Suelmann B.B.M., Mehra N., van Dijk N., Marsico G., van Montfoort M.L., Hackinger S., Braaf L.M., Amarante T. (2023). High- or low-dose preoperative ipilimumab plus nivolumab in stage III urothelial cancer: The phase 1B NABUCCO trial. Nat. Med..

[125] Nassar A.H., Umeton R., Kim J., Lundgren K., Harshman L., Van Allen E.M., Preston M., Dong F., Bellmunt J., Mouw K.W. (2019). Mutational Analysis of 472 Urothelial Carcinoma Across Grades and Anatomic Sites. Clin. Cancer Res..

[126] Guercio B.J., Sarfaty M., Teo M.Y., Ratna N., Duzgol C., Funt S.A., Lee C.-H., Aggen D.H., Regazzi A.M., Chen Z. (2023). Clinical and Genomic Landscape of FGFR3-Altered Urothelial Carcinoma and Treatment Outcomes with Erdafitinib: A Real-World Experience. Clin. Cancer Res..

[127] Sternberg C.N., Petrylak D.P., Bellmunt J., Nishiyama H., Necchi A., Gurney H., Lee J.L., van der Heijden M.S., Rosenbaum E., Penel N. (2023). FORT-1: Phase II/III Study of Rogaratinib Versus Chemotherapy in Patients with Locally Advanced or Metastatic Urothelial Carcinoma Selected Based on FGFR1/3 mRNA Expression. J. Clin. Oncol..

[128] Facchinetti F., Hollebecque A., Braye F., Vasseur D., Pradat Y., Bahleda R., Pobel C., Bigot L., Déas O., Florez Arango J.D. (2023). Resistance to Selective FGFR Inhibitors in FGFR-Driven Urothelial Cancer. Cancer Discov..

[129] Plimack E.R., Dunbrack R.L., Brennan T.A., Andrake M.D., Zhou Y., Serebriiskii I.G., Slifker M., Alpaugh K., Dulaimi E., Palma N. (2015). Defects in DNA Repair Genes Predict Response to Neoadjuvant Cisplatin-based Chemotherapy in Muscle-invasive Bladder Cancer. Eur. Urol..

[130] Galsky M.D., Wang H., Hahn N.M., Twardowski P., Pal S.K., Albany C., Fleming M.T., Starodub A., Hauke R.J., Yu M. (2018). Phase 2 Trial of Gemcitabine, Cisplatin, plus Ipilimumab in Patients with Metastatic Urothelial Cancer and Impact of DNA Damage Response Gene Mutations on Outcomes. Eur. Urol..

[131] Powles T., Carroll D., Chowdhury S., Gravis G., Joly F., Carles J., Fléchon A., Maroto P., Petrylak D., Rolland F. (2021). An adaptive, biomarker-directed platform study of durvalumab in combination with targeted therapies in advanced urothelial cancer. Nat. Med..

[132] Witjes J.A., Bruins H.M., Cathomas R., Compérat E.M., Cowan N.C., Gakis G., Hernández V., Linares Espinós E., Lorch A., Neuzillet Y. (2021). European Association of Urology Guidelines on Muscle-invasive and Metastatic Bladder Cancer: Summary of the 2020 Guidelines. Eur. Urol..

[133] Nawaf C., Shiang A., Chauhan P.S., Chaudhuri A.A., Agarwal G., Smith Z.L. (2023). Circulating tumor DNA based minimal residual disease detection and adjuvant treatment decision-making for muscle-invasive bladder cancer guided by modern clinical trials. Transl. Oncol..

[134] Carrasco R., Ingelmo-Torres M., Trullas R., Roldán F.L., Rodríguez-Carunchio L., Juez L., Sureda J., Alcaraz A., Mengual L., Izquierdo L. (2023). Tumor-Agnostic Circulating Tumor DNA Testing for Monitoring Muscle-Invasive Bladder Cancer. Int. J. Mol. Sci..

[135] Birkenkamp-Demtröder K., Christensen E., Nordentoft I., Knudsen M., Taber A., Høyer S., Lamy P., Agerbæk M., Jensen J.B., Dyrskjøt L. (2018). Monitoring Treatment Response and Metastatic Relapse in Advanced Bladder Cancer by Liquid Biopsy Analysis. Eur. Urol..

[136] Kovacs G., Akhtar M., Beckwith B.J., Bugert P., Cooper C.S., Delahunt B., Eble J.N., Fleming S., Ljungberg B., Medeiros L.J. (1997). The Heidelberg classification of renal cell tumours. J. Pathol..

[137] Sung H., Ferlay J., Siegel R.L., Laversanne M., Soerjomataram I., Jemal A., Bray F. (2021). Global Cancer Statistics 2020: GLOBOCAN Estimates of Incidence and Mortality Worldwide for 36 Cancers in 185 Countries. CA Cancer J. Clin..

[138] National Cancer Institute: Surveillance, E., and End Results Program. Cancer Stat Facts: Kidney and Renal Pelvis Cancer. https://seer.cancer.gov/statfacts/html/kidrp.html.

[139] Choueiri T.K., Motzer R.J. (2017). Systemic Therapy for Metastatic Renal-Cell Carcinoma. N. Engl. J. Med..

[140] Nuzzo P.V., Berchuck J.E., Korthauer K., Spisak S., Nassar A.H., Abou Alaiwi S., Chakravarthy A., Shen S.Y., Bakouny Z., Boccardo F. (2020). Detection of renal cell carcinoma using plasma and urine cell-free DNA methylomes. Nat. Med..

[141] Zill O.A., Banks K.C., Fairclough S.R., Mortimer S.A., Vowles J.V., Mokhtari R., Gandara D.R., Mack P.C., Odegaard J.I., Nagy R.J. (2018). The Landscape of Actionable Genomic Alterations in Cell-Free Circulating Tumor DNA from 21,807 Advanced Cancer Patients. Clin. Cancer Res..

[142] Kubiliute R., Jarmalaite S. (2021). Epigenetic Biomarkers of Renal Cell Carcinoma for Liquid Biopsy Tests. Int. J. Mol. Sci..

[143] Outeiro-Pinho G., Barros-Silva D., Aznar E., Sousa A.I., Vieira-Coimbra M., Oliveira J., Gonçalves C.S., Costa B.M., Junker K., Henrique R. (2020). MicroRNA-30a-5p(me): A novel diagnostic and prognostic biomarker for clear cell renal cell carcinoma in tissue and urine samples. J. Exp. Clin. Cancer Res..

[144] Lin Y.L., Wang Y.P., Li H.Z., Zhang X. (2017). Aberrant Promoter Methylation of PCDH17 (Protocadherin 17) in Serum and its Clinical Significance in Renal Cell Carcinoma. Med. Sci. Monit..

[145] Skrypkina I., Tsyba L., Onyshchenko K., Morderer D., Kashparova O., Nikolaienko O., Panasenko G., Vozianov S., Romanenko A., Rynditch A. (2016). Concentration and Methylation of Cell-Free DNA from Blood Plasma as Diagnostic Markers of Renal Cancer. Dis. Markers.

[146] Hoque M.O., Begum S., Topaloglu O., Jeronimo C., Mambo E., Westra W.H., Califano J.A., Sidransky D. (2004). Quantitative detection of promoter hypermethylation of multiple genes in the tumor, urine, and serum DNA of patients with renal cancer. Cancer Res..

[147] Perego R.A., Corizzato M., Brambilla P., Ferrero S., Bianchi C., Fasoli E., Signorini S., Torsello B., Invernizzi L., Bombelli S. (2008). Concentration and microsatellite status of plasma DNA for monitoring patients with renal carcinoma. Eur. J. Cancer.

[148] Mouliere F., Chandrananda D., Piskorz A.M., Moore E.K., Morris J., Ahlborn L.B., Mair R., Goranova T., Marass F., Heider K. (2018). Enhanced detection of circulating tumor DNA by fragment size analysis. Sci. Transl. Med..

[149] Bacon J.V.W., Annala M., Soleimani M., Lavoie J.M., So A., Gleave M.E., Fazli L., Wang G., Chi K.N., Kollmannsberger C.K. (2020). Plasma Circulating Tumor DNA and Clonal Hematopoiesis in Metastatic Renal Cell Carcinoma. Clin. Genitourin. Cancer.

[150] Yamamoto Y., Uemura M., Fujita M., Maejima K., Koh Y., Matsushita M., Nakano K., Hayashi Y., Wang C., Ishizuya Y. (2019). Clinical significance of the mutational landscape and fragmentation of circulating tumor DNA in renal cell carcinoma. Cancer Sci..

[151] Maia M.C., Bergerot P.G., Dizman N., Hsu J., Jones J., Lanman R.B., Banks K.C., Pal S.K. (2017). Association of Circulating Tumor DNA (ctDNA) Detection in Metastatic Renal Cell Carcinoma (mRCC) with Tumor Burden. Kidney Cancer.

[152] Chen W., Zhuang J., Wang P.P., Jiang J., Lin C., Zeng P., Liang Y., Zhang X., Dai Y., Diao H. (2019). DNA methylation-based classification and identification of renal cell carcinoma prognosis-subgroups. Cancer Cell Int..

[153] de Martino M., Klatte T., Haitel A., Marberger M. (2012). Serum cell-free DNA in renal cell carcinoma: A diagnostic and prognostic marker. Cancer.

[154] Jung M., Ellinger J., Gevensleben H., Syring I., Lüders C., de Vos L., Pützer S., Bootz F., Landsberg J., Kristiansen G. (2019). Cell-Free SHOX2 DNA Methylation in Blood as a Molecular Staging Parameter for Risk Stratification in Renal Cell Carcinoma Patients: A Prospective Observational Cohort Study. Clin. Chem..

[155] Hahn A.W., Gill D.M., Maughan B., Agarwal A., Arjyal L., Gupta S., Streeter J., Bailey E., Pal S.K., Agarwal N. (2017). Correlation of genomic alterations assessed by next-generation sequencing (NGS) of tumor tissue DNA and circulating tumor DNA (ctDNA) in metastatic renal cell carcinoma (mRCC): Potential clinical implications. Oncotarget.

[156] Khagi Y., Goodman A.M., Daniels G.A., Patel S.P., Sacco A.G., Randall J.M., Bazhenova L.A., Kurzrock R. (2017). Hypermutated Circulating Tumor DNA: Correlation with Response to Checkpoint Inhibitor-Based Immunotherapy. Clin. Cancer Res..

[157] Feng G., Ye X., Fang F., Pu C., Huang H., Li G. (2013). Quantification of plasma cell-free DNA in predicting therapeutic efficacy of sorafenib on metastatic clear cell renal cell carcinoma. Dis. Markers.

[158] Pal S.K., Sonpavde G., Agarwal N., Vogelzang N.J., Srinivas S., Haas N.B., Signoretti S., McGregor B.A., Jones J., Lanman R.B. (2017). Evolution of Circulating Tumor DNA Profile from First-line to Subsequent Therapy in Metastatic Renal Cell Carcinoma. Eur. Urol..

[159] Gonzalgo M.L., Eisenberger C.F., Lee S.M., Trock B.J., Marshall F.F., Hortopan S., Sidransky D., Schoenberg M.P. (2002). Prognostic Significance of Preoperative Molecular Serum Analysis in Renal Cancer. Clin. Cancer Res..

[160] Lasseter K., Nassar A.H., Hamieh L., Berchuck J.E., Nuzzo P.V., Korthauer K., Shinagare A.B., Ogorek B., McKay R., Thorner A.R. (2020). Plasma cell-free DNA variant analysis compared with methylated DNA analysis in renal cell carcinoma. Genet. Med..

[161] Wan J., Zhu L., Jiang Z., Cheng K. (2013). Monitoring of plasma cell-free DNA in predicting postoperative recurrence of clear cell renal cell carcinoma. Urol. Int..

[162] Chera B.S., Kumar S., Shen C., Amdur R., Dagan R., Green R., Goldman E., Weiss J., Grilley-Olson J., Patel S. (2020). Plasma Circulating Tumor HPV DNA for the Surveillance of Cancer Recurrence in HPV-Associated Oropharyngeal Cancer. J. Clin. Oncol..

[163] Naegele S., Efthymiou V., Das D., Sadow P.M., Richmon J.D., Iafrate A.J., Faden D.L. (2023). Detection and Monitoring of Circulating Tumor HPV DNA in HPV-Associated Sinonasal and Nasopharyngeal Cancers. JAMA Otolaryngol. Head Neck Surg..

[164] Balachandra S., Kusin S.B., Lee R., Blackwell J.M., Tiro J.A., Cowell L.G., Chiang C.M., Wu S.Y., Varma S., Rivera E.L. (2021). Blood-based biomarkers of human papillomavirus-associated cancers: A systematic review and meta-analysis. Cancer.

[165] Olesen T.B., Sand F.L., Rasmussen C.L., Albieri V., Toft B.G., Norrild B., Munk C., Kjær S.K. (2019). Prevalence of human papillomavirus DNA and p16(INK4a) in penile cancer and penile intraepithelial neoplasia: A systematic review and meta-analysis. Lancet Oncol..

[166] Ellinger J., Wittkamp V., Albers P., Perabo F.G.E., Mueller S.C., Ruecker A.v., Bastian P.J. (2009). Cell-Free Circulating DNA: Diagnostic Value in Patients with Testicular Germ Cell Cancer. J. Urol..

[167] Ellinger J., Albers P., Müller S.C., von Ruecker A., Bastian P.J. (2009). Circulating mitochondrial DNA in the serum of patients with testicular germ cell cancer as a novel noninvasive diagnostic biomarker. BJU Int..

[168] Ellinger J., Albers P., Perabo F.G., Müller S.C., von Ruecker A., Bastian P.J. (2009). CpG island hypermethylation of cell-free circulating serum DNA in patients with testicular cancer. J. Urol..

[169] Raos D., Oršolić D., Mašić S., Tomić M., Krasić J., Tomašković I., Gabaj N.N., Gelo N., Kaštelan Ž., Kuliš T. (2022). cfDNA methylation in liquid biopsies as potential testicular seminoma biomarker. Epigenomics.

[170] Nazha B., Zhuang T.Z., Dada H.I., Drusbosky L.M., Brown J.T., Ravindranathan D., Carthon B.C., Kucuk O., Goldman J., Master V.A. (2022). Blood-Based Next-Generation Sequencing in Adrenocortical Carcinoma. Oncologist.

[171] Raj N., Zheng Y., Kelly V., Katz S.S., Chou J., Do R.K.G., Capanu M., Zamarin D., Saltz L.B., Ariyan C.E. (2020). PD-1 Blockade in Advanced Adrenocortical Carcinoma. J. Clin. Oncol..

[172] Mota J.M., Sousa L.G., Braghiroli M.I., Siqueira L.T., Neto J.E.B., Chapchap P., Hoff A.A.O., Hoff P.M. (2018). Pembrolizumab for metastatic adrenocortical carcinoma with high mutational burden: Two case reports. Medicine.

[173] Garinet S., Nectoux J., Neou M., Pasmant E., Jouinot A., Sibony M., Orhant L., Pipoli da Fonseca J., Perlemoine K., Bricaire L. (2018). Detection and monitoring of circulating tumor DNA in adrenocortical carcinoma. Endocr.-Relat. Cancer.

[174] Fiala C., Diamandis E.P. (2018). Utility of circulating tumor DNA in cancer diagnostics with emphasis on early detection. BMC Med..

[175] Esfahani M.S., Hamilton E.G., Mehrmohamadi M., Nabet B.Y., Alig S.K., King D.A., Steen C.B., Macaulay C.W., Schultz A., Nesselbush M.C. (2022). Inferring gene expression from cell-free DNA fragmentation profiles. Nat. Biotechnol..

[176] Cristiano S., Leal A., Phallen J., Fiksel J., Adleff V., Bruhm D.C., Jensen S., Medina J.E., Hruban C., White J.R. (2019). Genome-wide cell-free DNA fragmentation in patients with cancer. Nature.

[177] Abbosh C., Birkbak N.J., Wilson G.A., Jamal-Hanjani M., Constantin T., Salari R., Le Quesne J., Moore D.A., Veeriah S., Rosenthal R. (2017). Phylogenetic ctDNA analysis depicts early-stage lung cancer evolution. Nature.

[178] Merker J.D., Oxnard G.R., Compton C., Diehn M., Hurley P., Lazar A.J., Lindeman N., Lockwood C.M., Rai A.J., Schilsky R.L. (2018). Circulating Tumor DNA Analysis in Patients with Cancer: American Society of Clinical Oncology and College of American Pathologists Joint Review. J. Clin. Oncol..

